# Early boosting of p38 MAPK signaling pathway by lycorine hydrochloride potently inhibits PRRSV proliferation in primary and established cells

**DOI:** 10.3389/fmicb.2025.1664973

**Published:** 2025-08-21

**Authors:** Lizhan Su, Zhening Zhang, Siqi Zhang, Jiajing He, Yaoqi Chen, Guohong Wu, Zhiying Zhang, Weisan Chen, Mingxin Zhang, Jianxin Chen

**Affiliations:** ^1^Guangdong Provincial Key Laboratory of Veterinary Pharmaceutics Development and Safety Evaluation, College of Veterinary Medicine, South China Agricultural University, Guangzhou, China; ^2^Department of Biochemistry and Genetics, La Trobe Institute for Molecular Science, La Trobe University, Melbourne, VIC, Australia; ^3^Southern China Institute of Large Animal Models for Biomedicine, School of Pharmacy and Food Engineering, Wuyi University, Jiangmen, China

**Keywords:** porcine reproductive and respiratory syndrome virus (PRRSV), lycorine hydrochloride (LH), p38 mitogen-activated protein kinase (p38 MAPK), NOD-, LRR- and pyrin domain-containing protein 3 (NLRP3), interleukin-1β (IL-1β)

## Abstract

Porcine reproductive and respiratory syndrome virus (PRRSV) has caused tremendous economic losses in the swine industry since emerging in the late 1980s. Although vaccination has been widely used to control PRRS epidemics in Chinese pig farms, they provided limited protection against PRRSV transmission; moreover, no effective therapeutic drugs are available. Therefore, there is an urgent need to develop novel antiviral strategies to control PRRSV epidemics. This study showed that Lycorine hydrochloride (LH), an isoquinoline alkaloid isolated from the bulb of *Lycoris radiata* herb, potently suppressed PRRSV replication in Marc-145 cells and primary porcine alveolar macrophages *ex vivo* (PAMs) with 50% effective antiviral concentrations (EC_50_) less than 1 μM. LH exhibited broad-spectrum inhibitory activities *in vitro* against clinically circulating type 2 PRRSV GD-HD, CH-1a, and NADC30-like HNhx strains in China. Mechanistically, LH treatment induced a rapid up-regulation of p38 MAPK signaling pathway in both infected and uninfected cells, which enhanced the expressions of NLRP3 and Caspase-1 proteins, thereby promoting the conversion of pro-IL-1β to IL-1β, which led to decreased PRRSV replication. In summary, this study reveals that early boosting of p38 MAPK signaling pathway induced by LH treatment significantly suppresses PRRSV propagation, and LH has the potential to be used as a novel antiviral agent against PRRSV infections.

## Introduction

Porcine reproductive and respiratory syndrome virus (PRRSV) is a highly contagious swine virus that causes enormous economic losses in pig production globally (Neumann et al., 2005). The primary symptoms of PRRSV infection include severe reproductive failure in pregnant sows, as well as respiratory disorders in piglets and growing pigs (Zhang et al., 2017). PRRSV is an enveloped, single-stranded, positive-sense RNA virus that belongs to the genus *Porartevirus*, the family *Arteriviridae*, and the order *Nidovirales* (Kuhn et al., 2016). The entire length of PRRSV genome is approximately 15.4 kb, which contains a 5′-cap and 3′-poly A tail structures (Dokland, 2010). Currently, vaccination is the primary method for controlling the spread of the virus within swine populations. The primary vaccines used in clinical settings are modified live and inactivated virus vaccines. However, modified live virus vaccines carry the risk of virulence reversion, while inactivated virus vaccines often exhibit insufficient immunogenicity (Charerntantanakul, 2012). Consequently, the protection offered by these vaccines against PRRSV infections is often limited. Therefore, developing antiviral drugs against PRRSV is urgently needed. Previous studies have identified some natural compounds with anti-PRRSV activity, including Matrine (Sun et al., 2019), artesunate (Long et al., 2022), and (−)-epigallocatechin-3-gallate (Ge et al., 2018). While most of these compounds have shown potential in inhibiting PRRSV replication in cell cultures, their antiviral efficacy *in vivo* and in clinical applications has yet to be demonstrated.

Viral replication is closely intertwined with many host cell processes, most of them involving host cell kinases. These kinases play pivotal roles in regulating viral replication by modulating various intracellular signaling pathways. They not only influence viral entry and replication but also shape the host immune response, making them critical targets for developing antiviral drugs (Ludwig et al., 2002; Li et al., 2015). Mitogen-activated protein kinases (MAPKs) are members of cellular kinase family that play critical roles in cell signaling and gene expression (Rezatabar et al., 2019). The mammalian MAPKs family includes c-Jun N-terminal kinases (JNK), p38, and extracellular signal-regulated kinases (ERK). These three kinases interact with each other to exert their unique biological functions (Roux and Blenis, 2004; Shaul and Seger, 2007). Previous studies have indicated that the MAPK signaling pathway is closely associated with PRRSV replication. During the late stages of PRRSV infection, JNK, p38 and ERK kinase activities are significantly enhanced, which facilitates PRRSV replication in immortalized porcine alveolar macrophage and Marc-145 cells (Lee and Lee, 2012; Wang et al., 2023). However, during the early stages of PRRSV infection, JNK and ERK exhibit distinct roles. During the initial infection, PRRSV attenuates JNK phosphorylation and suppresses apoptosis to facilitate its replication (Huo et al., 2013). In contrast, p-ERK is increased during this time to promote viral entry (Lee and Lee, 2010). p-p38 increases in the late stages of PRRSV infection and primarily regulates the expression of cytokines induced by PRRSV infection, such as IL-1β (Bi et al., 2014), IL-10 (Song et al., 2013), and IL-17 (Wang et al., 2019). PRRSV infection induces the productions of miRNA novel-216 via the p38 signaling pathway, leading to inhibited expression of type I IFN and IFN-stimulated genes and enhanced viral replication (Luo et al., 2024). These findings indicated that PRRSV infection induces a late activation of p38 MAPK pathway, and in return increased p-p38 promotes PRRSV replication. However, how early increased p-p38 promotes PRRSV propagation remains unclear.

p38 MAPK exerts crucial functions in various inflammatory responses. The activation of p38 MAPK mediates the production of pro-inflammatory cytokines such as TNF-*α*, IL-1β, and IL-6, as well as the overall inflammatory response (Ishijima and Nakajima, 2021). In the real scene, there is significant crosstalk between the p38 MAPK signaling pathway and other inflammatory signaling pathways, such as nuclear factor-kappa B (NF-κB) pathway and NOD-, LRR- and pyrin domain-containing protein 3 (NLRP3) inflammasome pathway (Li et al., 2018). NLPR3 inflammasome is composed of NLPR3, an adaptor protein apoptosis-associated speck-like protein (ASC) and Caspase-1, and it is important for inflammatory response. Activation of the NLPR3 inflammasome could modulate the activation of Caspase-1, resulting in the conversion of pro-IL-1β into IL-1β. The production and release of pro-inflammatory cytokines is one of the fundamental reactions of cellular innate immunity to viral infection (Kelley et al., 2019). IL-1β plays a critical role in the inflammatory response against pathogens. Previous studies have demonstrated that IL-1β possesses potent antiviral effects against various viral infections, including hepatitis B virus (HBV) (Isorce et al., 2016), influenza A virus (IAV) (Schmitz et al., 2005), African swine fever virus (ASFV) (Li J. et al., 2021). Our previous research showed that toosendanin, a natural tetracyclic triterpenoid from plants, induces IL-1β maturation by activating innate immune sensor IFI16, thereby indirectly inhibiting PRRSV replication (Zhang et al., 2018). However, little is reported about developing IL-1β inducer as antiviral agents.

Lycorine is an isoquinoline alkaloid isolated from the bulb of *Lycoris radiata* herb. Accumulated studies have shown that lycorine possess diverse pharmacological activities including anti-tumoral, anti-inflammatory, anti-bacterial and anti-parasitic effects (Cao et al., 2013), as well as fibrosis inhibition (Wu et al., 2021) and efficacy in Parkinson’s disease treatment (Zhu et al., 2021). Moreover, lycorine was reported to have antiviral activities, such as IAV, dengue virus (DENV), alphaviruses and SARS-CoV-2 (Zou et al., 2009; He et al., 2013; Zhang et al., 2020; Li N. et al., 2021). Lycorine hydrochloride (LH), the hydrochloride salt form of lycorine, was used as an alternative in many studies due to its better water solubility and bioavailability than lycorine. So far, there is no report on anti-PRRSV effects of lycorine and LH. In the present study, we identified LH as a potent anti-PRRSV agent in Marc-145 cells and PAMs with 50% effective antiviral concentrations (EC_50_) less than 1 μM. Furthermore, we demonstrated that LH’s inhibition on PRRSV infection is associated with its up-regulation of p-p38 and the activation of downstream NLRP3/Caspase-1/IL-1β pathway. To our knowledge, this is the first report for LH’s anti-PPRSV activity and its mechanisms.

## Materials and methods

### Compounds and reagents

Lycorine hydrochloride (LH) and ribavirin (Rib) were purchased from Chengdu Pufeide Biotechnology Co., Ltd. (Chengdu, China) and Star Lake Bioscience Co., Ltd. (Zhaoqing, China) with certified purity ≥99.5%, respectively. LH and Rib stock solutions were prepared in dimethyl sulfoxide (DMSO; Sigma-Aldrich, MO, United States) and subsequently diluted in DMEM prior to be used for assays. Final working concentrations of DMSO in all treatment groups were controlled below 0.4%. Recombinant porcine Interleukin-1β (rmIL-1β) was purchased from Beyotime Biotechnology (Shanghai, China). 3-(4,5-dimethylthiozol-2-yl)-3,5-dipheryl 640 tetrazolium bromide (MTT) was purchased from Sigma-Aldrich (MO, United States).

### Cell lines and viruses

Marc-145 cells, a PRRSV-permissive cell line derived from African green monkey kidney cell line MA-104, were obtained from the American Type Culture Collection (ATCC) and grown in Dulbecco’s minimum essential medium (DMEM, Gibco, UT, United States) supplemented with 10% fetal bovine serum (FBS, Biological Industries, Kibbutz Beit HaEmek, Israel) and 100 IU/mL of penicillin and 100 μg/mL streptomycin at 37°C with 5% CO2.

Porcine alveolar macrophages (PAMs) were prepared as described in previous methods (Brockmeier et al., 2017). In brief, PAMs were obtained from lung lavage fluid of healthy 4-week-old Large-White piglets with negative PCR and antibody test strip PRRSV and Porcine circovirus type 2 (PCV2) results. Following 3 washes with PBS, cells were centrifuged at 800 g for 10 min. The PAMs were resuspended in RPMI 1640 supplemented with 10% FBS and 100 IU/ mL of penicillin and 100 μg/mL streptomycin at 1 × 10^6^ cells/mL in 6-well plate, and then incubated at 37°C for 2 h.

Three Type 2 PRRSV strains, GD-HD (GenBank ID: KP793736), CH-1a (GenBank ID: AY032626), and NADC30-like HNhx (GenBank ID: KX766379) strains (Xie et al., 2013), were propagated in Marc-145 cells in DMEM with 3% FBS (essential medium). Virus preparations were stored at −80°C. Virus titers were determined using a microtitration infectivity assay (Kim et al., 1993).

### Cytotoxicity assay

The cytotoxicity of LH was assessed using MTT assay (Ge et al., 2018). Briefly, Marc-145 cells (5 × 10^4^ cells/well) or PAMs (2 × 10^5^ cells/well) were seeded in 96-well plates and incubated at 37°C for 24 h and 6 h, respectively. The medium was then replaced with fresh medium containing serial dilutions of LH, followed by incubation for 48 h.

The culture medium was removed and replaced with 100 μL 3-(4,5-dimethylthiozol-2-yl)-3,5-dipheryl tetrazolium bromide (MTT, Sigma-Aldrich, Saint Louis, United States) solution (0.5 mg/mL in PBS) and incubated at 37°C for 4 h. After removing the supernatant, 150 μL of DMSO was added for 10 min at 37°C to dissolve the formazan crystals. The cell viability was determined using a microplate reader, measuring the absorbance at 490 nm (Thermo Fisher scientific, MA, United States). The mean optical density (OD) values from six wells per treatment were used as the cell viability indexes. The 50% cytotoxic concentration (CC_50_) was analyzed using GraphPad Prism 7.0 (GraphPad Software, San Diego, CA, United States).

### Indirect immunofluorescence assay (IFA)

Immunofluorescence analysis was performed on PRRSV-infected and mock-infected cell cultures through sequential antigen retrieval steps. Cells were subjected to sequential fixation (4% paraformaldehyde, 10 min) followed by membrane permeabilization [0.25% Triton X-100, 10 min at room temperature (RT)] and nonspecific binding blockade (3% BSA, 60 min, RT). Primary antibody incubation employed murine-derived monoclonal antibody against the N protein of PRRSV (clone 4A5, 1:800; MEDIAN Diagnostics, Korea) under refrigeration conditions (4°C, 16 h). Following PBS washes, antigen–antibody complexes were visualized using species-matched Alexa Fluor 568-conjugated secondary antibody (1:1000, Cell Signaling Technology, MA, USA) through 60 min RT incubation. Nuclear counterstaining was achieved with DAPI (300 nM, Sigma-Aldrich) prior to multispectral imaging using a Leica DMI 4000B fluorescence imaging system (Leica Microsystems, Germany). Quantitative analysis involved enumerating DAPI-positive nuclei (total cells) and Alexa Fluor 568-positive signals (infected cells) across microscopic fields. Infection frequency was calculated as (infected cells/total cells) × 100%, with therapeutic efficacy normalized against vehicle control (DMSO-treated) groups. The 50% effective concentration (EC_50_) for viral inhibition was derived through nonlinear dose–response modeling of compound activity profiles using GraphPad Prism 7.0.

### Real-time quantitative reverse-transcription PCR (qRT-PCR)

Total RNA was extracted using the total RNA rapid extraction kit (Fastagen, Shanghai, China) following the manufacturer’s instructions. RNA was reverse-transcribed into first-strand cDNA using a reverse-transcription kit (Genstar, Beijing, China). The primers used for PCR amplification are listed in Table 1 (Zhang et al., 2022). A 2 Real Star Green Power Mixture (including SYBR Green I Dye) (Genstar, Beijing, China) was used to perform real-time quantitative reverse transcription PCR (qRT-PCR) (Bio-Rad, California, United States) using the CFX96 Real-time PCR machine. PCR amplification was performed on 1 μL of reverse transcribed product with primers designed against PRRSV-Nsp9, IL-1β, and GAPDH (glyceraldehyde-3-phosphate dehydrogenase; used as the endogenous control).

**Table 1:** Primer sequences for qRT-PCR.

Name	Sequences 5′ to 3’
Nsp9-F	5’- CTAAGAGAGGTGGCCTGTCG −3’
Nsp9-R	5’- GAGACTCGGCATACAGCACA −3’
mGAPDH-F	5’- GCAAAGACTGAACCCACTAATTT −3′
mGAPDH-R	5’-TGTCATCATATTTGGCAGGTTT-3’
pGAPDH-R	5’- TTGCCTCTGTTGTTACTTGGAGAT −3′
pGAPDH-F	5’-TGCCAACGTGTCGGTTGT-3’
IL-1β-F	5’- CCCAAAAGTTACCCGAAGAGG −3′
IL-1β-R	5′- TCTGCTTGAGAGGTGCTGATG −3’

### Enzyme-linked immunosorbent assay (ELISA)

The ELISA kit was used to determine the level of IL-1β secreted in the cell supernatant. AiFang Biotechnology Company (Hunan, China) provided the kit (Pig. NO. AF-01256P1). The whole examination was carried out according to the instructions.

### Western blotting analysis

Cellular lysates were prepared using RIPA lysis buffer (Beyotime, China) containing protease/phosphatase inhibitor cocktail, with equal protein quantities resolved through denaturing SDS-PAGE and transferred to polyvinylidene-fluoride (PVDF) membranes (Millipore, United States) via semi-dry electrophoretic transfer. Membranes underwent sequential incubations with primary antibodies at 1:1000 dilution in blocking buffer at 4°C for 16 h, including: murine-derived anti-PRRSV N protein (4A5 clone, MEDIAN Diagnostics, Korea); rabbit monoclonals from Cell Signaling Technology (United States) targeting NLRP3 (15101), p38 MAPK (9212), phospho-p38 MAPK (Thr180/Tyr182) (9211), Caspase-1 (24232), and IL-1β (12703); along with *α*-Tubulin (Beyotime, China), DDDDK-Tag (ABclonal, China), and GAPDH (GoodHere, China). Following species-appropriate HRP-conjugated secondary antibody incubations (1 h, RT), protein-antibody complexes were visualized through chemiluminescent detection using enhanced ECL substrates, with signal acquisition performed on a ChemiDoc MP imaging system (Bio-Rad, United States). GAPDH and α-Tubulin were used as loading controls, with the selection of either as the internal reference in individual experiments determined by the adequacy of electrophoretic separation between target protein bands and reference protein bands.

### Antiviral activity assay

Marc-145 cells or PAMs in 96-well plates were infected with PRRSV (100 or 1,000 TCID_50_) in essential medium for 2 h at 37°C. The culture supernatants were then removed and fresh DMEM containing different concentrations of each compound was added. Cells and supernatants were then collected at the indicated time points, and the virus titer and viral RNA level were determined by the endpoint dilution assay and qRT-PCR, respectively. Cells were subjected to PRRSV-infected cell counting by IFA, and their viral NP level was determined by Western blotting.

### Time-of-addition assay

For pretreatment, Marc-145 cells were pretreated with LH in essential medium for 2 or 4 h at 37°C. After three washes with PBS, cells were infected with PRRSV for 2 h and collected at 24 h post-infection (hpi) for IFA. For co-treatment, the cells were simultaneously incubated with PRRSV and LH at 37°C. After 2 h, the mixture was removed, and the cells were washed three times with PBS before adding fresh medium. At 24 hpi, the samples were submitted to analysis.

### Gene silencing with siRNA

The specific siRNAs targeting p38 (sip38) and the control siRNA (siNC) were designed and synthesized by Sangon Biotech (Shanghai, China). The siRNA sequences are as follows: sip38-396, 5-UAGACCUCGGAGAAUUUGGTT-3′; sip38-450, 5′-UAGAUUACUAGGUUUUAGGTT-3′; sip38-728, 5′-UUCUUCAAAAGCUCAGCCCTT-3′; the nontargeting control siRNA (siNC), 5′-UUCUCCGAACGUGUCACGUTT-3′. The p38 siRNA and negative control siRNA were transfected into Marc-145 cells for 24 h using Lipofectamine^™^ 3,000 Transfection Reagent (Thermo Scientific, CA, United States), 24 h post-transfection (hpt), the transfected cells were infected with PRRSV and treated with various concentrations of LH, followed by further analysis.

### Plasmid construction

The full-length sequence of the p38 (GeneID: 103221596) cDNA was obtained using PCR and the gene was cloned into the mammalian expression vector pCAG-MCS-3xFLAG. All specific primers used for plasmid construction were designed using Primer Premier 5. Lipofectamine^™^ 3,000 was used to transfect plasmids into Marc-145 cells. For protein overexpression, Marc-145 cells were transfected for 24 h before harvest. The expression of Flag-p38 was detected using Western blotting.

### Statistical analysis

All experiments were performed at least three times. The results were presented as mean standard deviation (SD). Statistical significance was determined by the Student’s t test when only two groups were compared or by one-way analysis of variance (ANOVA) when more than two groups were compared. *p < 0.05, **p < 0.01, and ***p < 0.001 were considered to be statistically significant at different levels.

## Results

### LH inhibits PRRSV infection in Marc-145 cells and PAMs

**Figure 1:**
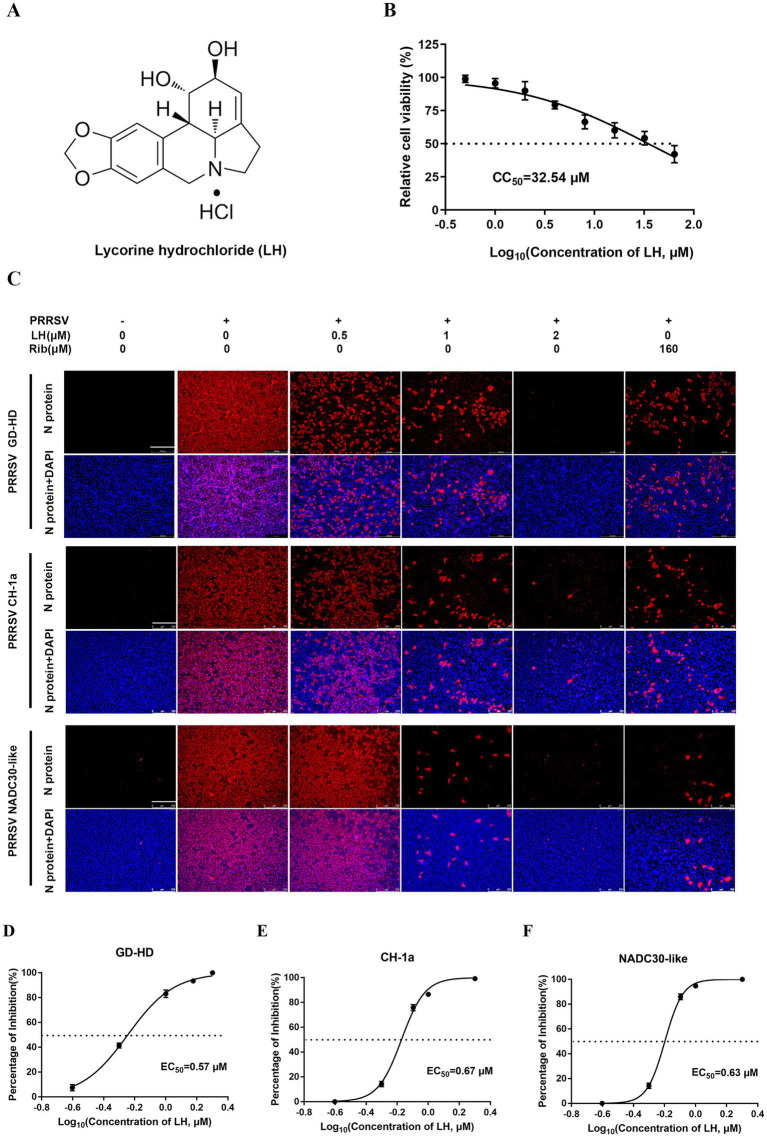
Cytotoxicity and anti-PRRSV activity of lycorine hydrochloride (LH) in Marc-145 cells. **(A)** Chemical structure of LH. **(B)** Cytotoxicity of LH on Marc-145 cells was evaluated using the MTT assay, and the percentage of cellular viability in the absence of the compound was set to 100. **(C)** Antiviral activities of LH against three different PRRSV strains (GD-HD, CH-1a, and NADC30-like) were examined by immunofluorescence assays (IFA). The cells were infected with PRRSV (100 TCID_50_) at 37°C for 2 h and then treated with fresh medium containing different concentrations of LH (0.5, 1 or 2 μM) or ribavirin (Rib, 160 μM) for 48 h. The antibody against N protein (NP) was used to stain PRRSV NP, followed by the Alexa Fluor 568-conjugated goat anti-mouse secondary antibody (red). Cellular nuclei were counterstained using 4,6-diamidino-2-phenylindole (DAPI) (blue). **(D–F)** The percentage of reduced PRRSV-infected cell number in IFA images was used to determine the concentration required to protect 50% cells from PRRSV infection (EC_50_).

The chemical structure of LH is shown in Figure 1A. The cytotoxicity of LH on Marc-145 cells was tested by MTT assays. As shown in Figure 1B, the half cytotoxic concentration (CC_50_) of LH on Marc-145 cells is calculated as 32.54 μM using GraphPad Prism 7.0 software. Next, the antiviral activity of LH was examined against different PRRSV strains (GD-HD, CH-1a, and NADC30-like) by immunofluorescence assays (IFA). As shown in Figures 1C–F, LH significantly inhibited the replication of PRRSV GD-HD, CH-1a, and NADC30-like in Marc-145 cells in a dose-dependent manner. The 50 % effective concentration (EC_50_) values of LH against the infections by the three tested PRRSV strains were determined to range from 0.57 to 0.63 μM by quantifying infected cells from IFA images, which were significantly lower than the EC_50_ value of Rib (138.1 μM) (Supplementary Figure S1). The antiviral selectivity index of LH (SI, CC_50_/EC_50_) ranged from 48.5 to 57.1 (Table 2).

**Table 2:** Inhibitory activity of LH against infections by different PRRSV strains in Marc-145 cells.

EC_50_ and SI value	PRRSV strains		
	GD-HD	CH-1a	NADC30-like
EC_50_ (μM)^a^	0.57 ± 0.01	0.67 ± 0.02	0.63 ± 0.01
Selectivity index (SI)^b^	57.1 ± 1.0	48.5 ± 1.4	51.6 ± 0.83

a EC_50_, the concentration required to protect 50% cells from PRRSV infection by counting infected cells from IFA images, as described in the methods.

b SI (selectivity index) is the ratio of CC_50_ to EC_50_. CC_50_ of LH on Marc-145 cells was 32.54 μM.

**Figure 2:**
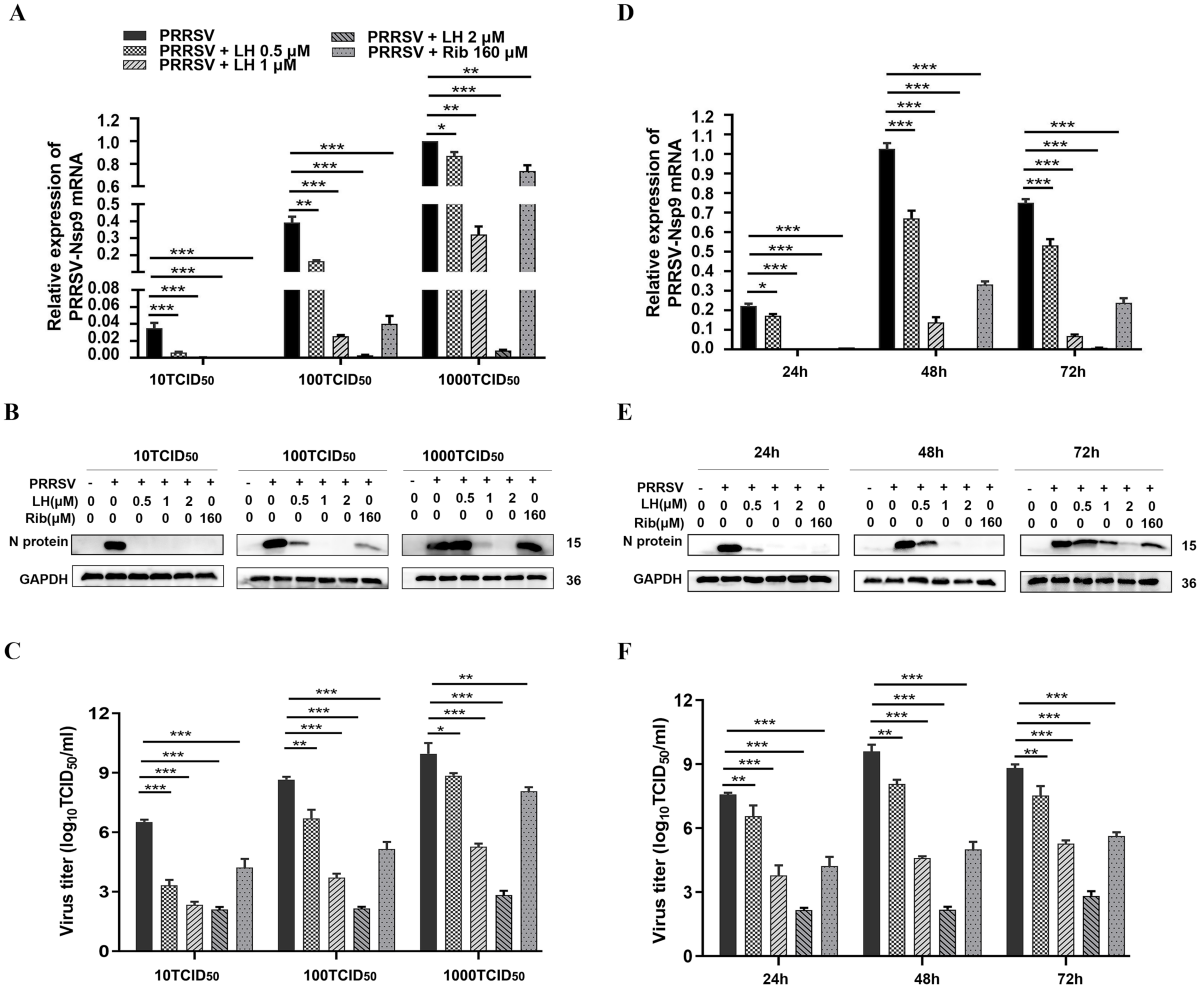
LH inhibits PRRSV infection in Marc-145 cells. **(A–C)** Cells grown in 12-well plates were infected with PRRSV GD-HD (10, 100 or 1,000 TCID_50_) for 2 h at 37°C and then cultured in fresh medium containing various concentrations of LH (0.5, 1 and 2 μM) or Rib (160 μM). At 48 hpi, the samples were collected and analyzed by. **(D–F)** Cells grown in 12-well plates were infected with PRRSV GD-HD (100 TCID_50_) for 2 h at 37°C and then cultured in fresh medium containing various concentrations of LH (0.5, 1 and 2 μM) or Rib (160 μM). At the indicated time points post-infection, the samples were collected and analyzed by qRT-PCR **(D)**, Western blotting **(E)** and viral titer titration **(F)**. Statistical significances are denoted by *p < 0.05, **p < 0.01, and ***p < 0.001, compared with the DMSO control (only PRRSV infection).

Furthermore, we examined the antiviral effects of LH against different infection doses (10, 100 and 1,000 TCID_50_) of GD-HD strain using qRT-PCR, Western blotting, and viral titer titration. As shown in Figures 2A,B, LH significantly suppressed viral Nsp9 mRNA (Figure 2A) and N protein (Figure 2B) levels in PRRSV infected Marc-145 cells. Meanwhile, LH treatment effectively reduced the virus titer in a dose-dependent manner (Figure 2C). LH treatment at 2 μM led to reduced progeny virus production by 4.5, 6.4, and 7.2 logs compared to the DMSO controls for the three viral infection doses (10, 100 and 1,000 TCID_50_) (Figure 2C), respectively. In addition, we evaluated whether LH has a sustained inhibitory effect on PRRSV replication by analyzing the dynamic levels of viral RNA, protein expression and virus titer from 24 to 72 hpi. LH treatment (0.5, 1, and 2 μM) significantly reduced the levels of viral Nsp9 mRNA, N protein expression, and virus titer at three time-points (24, 48 and 72 hpi), and LH (1 and 2 μM) showed a superior antiviral effect compared to the positive control Rib (160 μM) (Figures 2D–F). Collectively, these results demonstrate that LH significantly inhibits PRRSV replication in Marc-145 cells.

**Figure 3:**
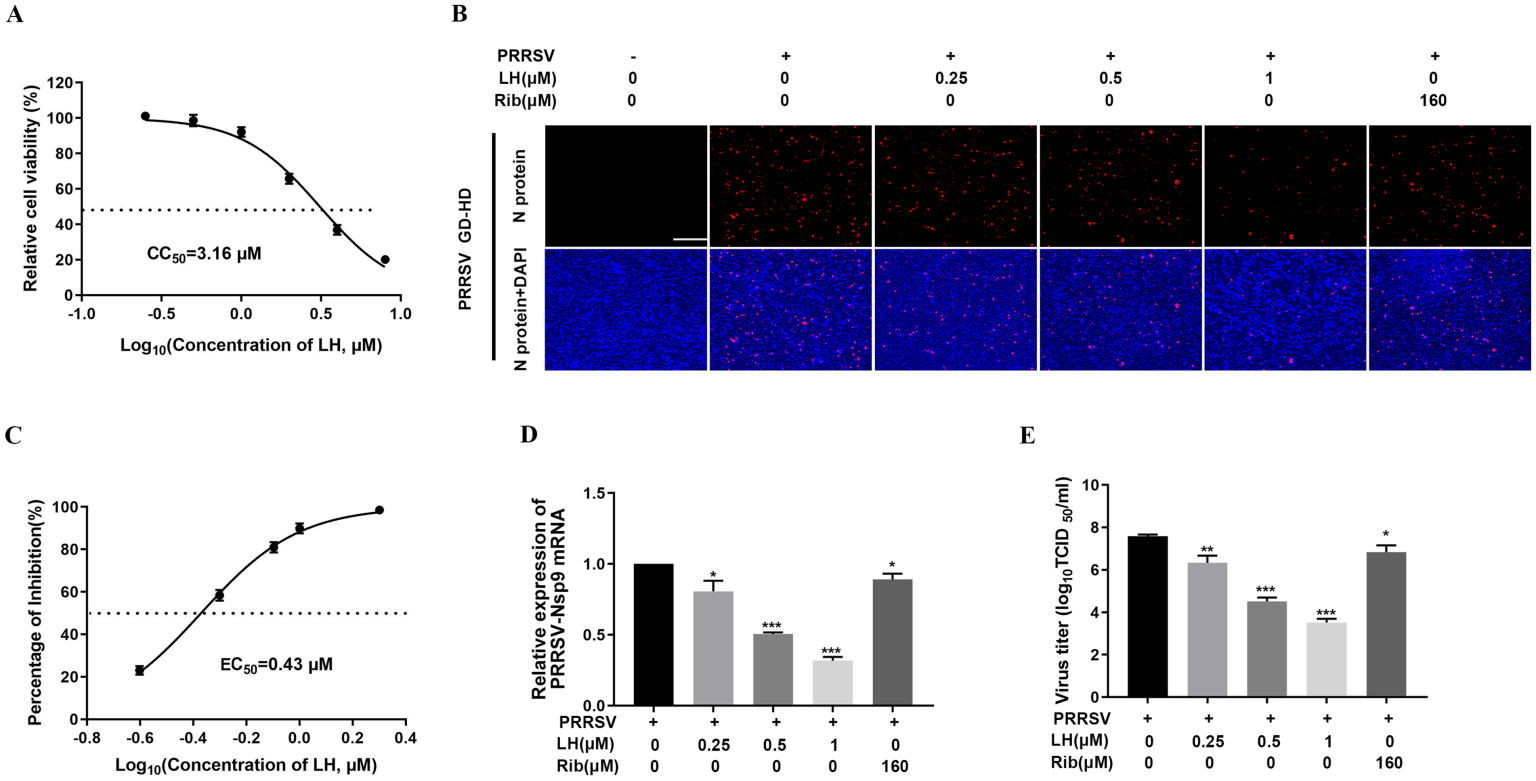
LH inhibits PRRSV infection in PAMs. **(A)** Cytotoxicity of LH in PAMs was examined using a MTT assay as described in the methods. PAMs cultured in 96-well plates **(B,C)** or 12-well plates **(D,E)** were infected with PRRSV GD-HD (1,000 TCID_50_) for 2 h at 37°C and then treated with LH at various concentrations. At 24 hpi, the samples were subjected to IFA **(B)**, qRT-PCR **(D)** and viral titer titration **(E)** analyses. **(B)** Representative IFA images are extracted from three independent experiments. **(C)** The percentage of reduced PRRSV-infected cell number in IFA images was used to determine the concentration required to protect 50% cells from PRRSV infection (EC_50_). Statistical significances are denoted by *p < 0.05, **p < 0.01, and ***p < 0.001, compared with the DMSO control.

Given that PAMs are the major target cells of PRRSV infection in pigs, we investigated whether LH could inhibit PRRSV replication in PAMs *ex vivo*. The CC_50_ value of LH in PAMs was measured by MTT assays and calculated to be 3.16 μM (Figure 3A). The anti-PRRSV activity of LH was then assessed by determining the viral mRNA and N protein levels, as well as virus titers. LH treatment (0.25, 0.5, and 1 μM) significantly suppressed PRRSV replication (Figure 3B), and the EC_50_ of LH against GD-HD PRRSV replication in PAMs was 0.43 μM (Figure 3C). Similarly, LH treatments resulted in a significant reduction in viral Nsp9 mRNA levels (Figure 3D) and virus titers (Figure 3E) in a dose-dependent manner. These results demonstrate that LH significantly inhibits PRRSV replication in PAMs.

### LH suppresses PRRSV infection in pre-treatment and co-treatment modes

**Figure 4:**
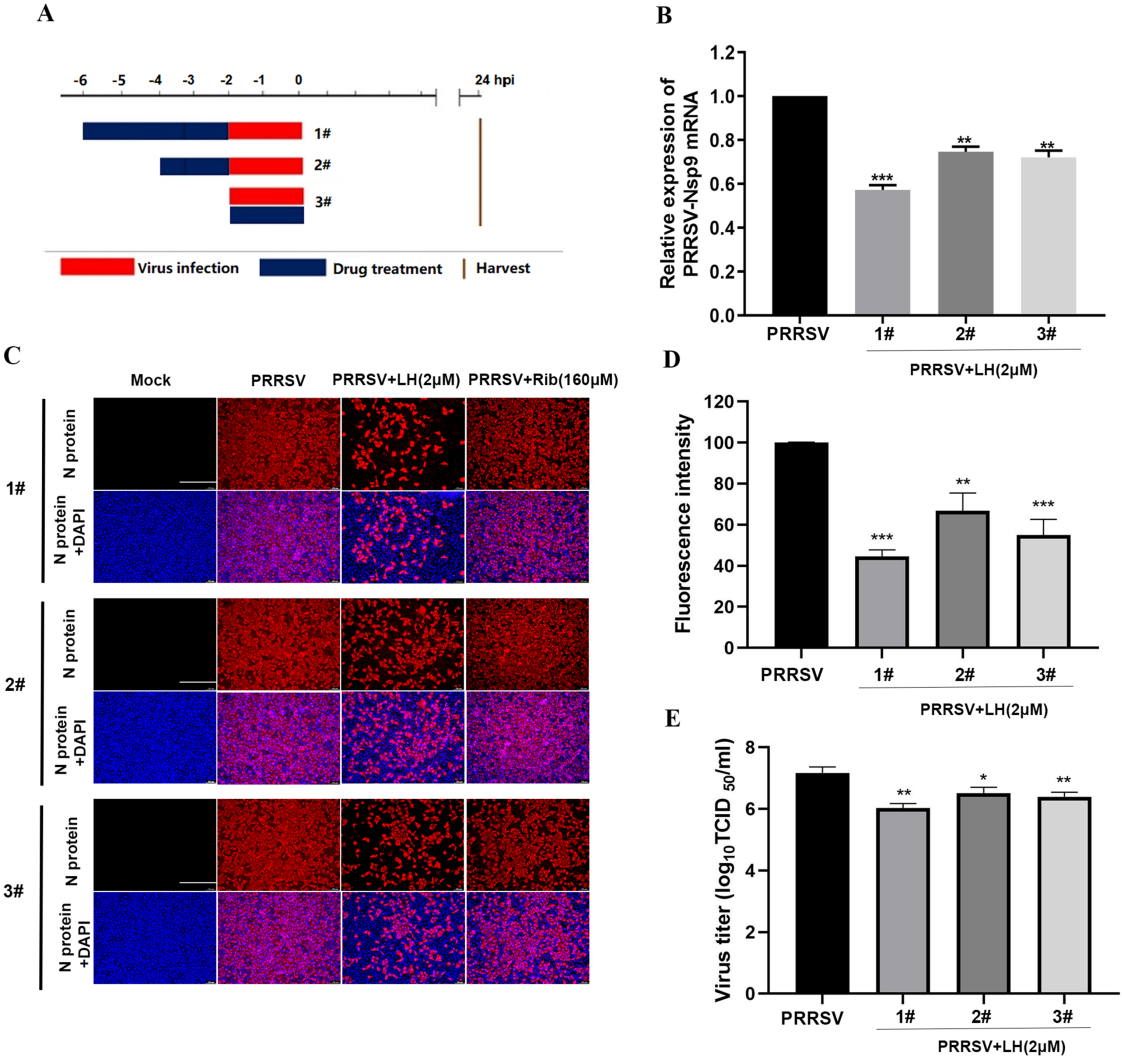
LH suppresses PRRSV infection in pre- and co-treatment modes. For pre-treatment, Marc-145 cells were pretreated with LH in essential medium for 2 or 4 h at 37°C. After three washes with PBS, cells were infected with PRRSV for 2 h. For co-treatment, the cells were simultaneously incubated with PRRSV and LH at 37°C. After 2 h, the mixture was removed, and the cells were washed three times with PBS before adding fresh medium, as shown in the schedules **(A)**. At 24 hpi, the samples were subjected to qRT-PCR **(B)**, IFA **(C,D)** and viral titer titration **(E)** analysis. Representative IFA images **(C)** are extracted from three independent experiments, and the intensity of red fluorescence in IFA images was calculated using Image J software. Statistical significances are denoted by *p < 0.05, **p < 0.01, and ***p < 0.001, compared with the DMSO control.

To initially explore the antiviral mechanisms of LH, the Marc-145 cells were infected with PRRSV for 2 h, and LH was added to the cell cultures before (pre-treatment) or during (co-treatment) PRRSV infection for different incubation periods (Figure 4A). As shown in Figures 4B–E, LH treatment significantly inhibited viral mRNA levels, viral NP expressions and virus titers in the pre-treatment mode, and the inhibitory effect was correlated with the incubation time of LH. Moreover, when LH was added to the cells during PRRSV infection (co-treatment), there was a significant suppression of viral mRNA levels, viral NP expression and virus titers. These results collectively indicated that LH has a substantial suppressive effect on PRRSV replication in Marc-145 cells when treated prior to or during viral infection, indicating cellular protein or process is likely affected.

To exclude the possibility that LH might exert its antiviral effect by direct PRRSV interaction, PRRSV GD-HD (1,600 TCID_50_) and 2 μM of LH were mixed in essential medium and incubated for 2 h at 37°C. The mixture was then diluted by 16-fold, and the diluted mixture (containing 100 TCID_50_ PRRSV and 0.125 μM LH) was used to infect Marc-145 cells for 2 h (Supplementary Figure S2A). At 48 hpi, the samples were analyzed by IFA. As shown in Supplementary Figure S2B, incubation of LH with the virus did not affect viral N protein expression, suggesting that LH does not target the PRRSV virions.

### LH induces p38 phosphorylation in Marc-145 cells and PAMs

**Figure 5:**
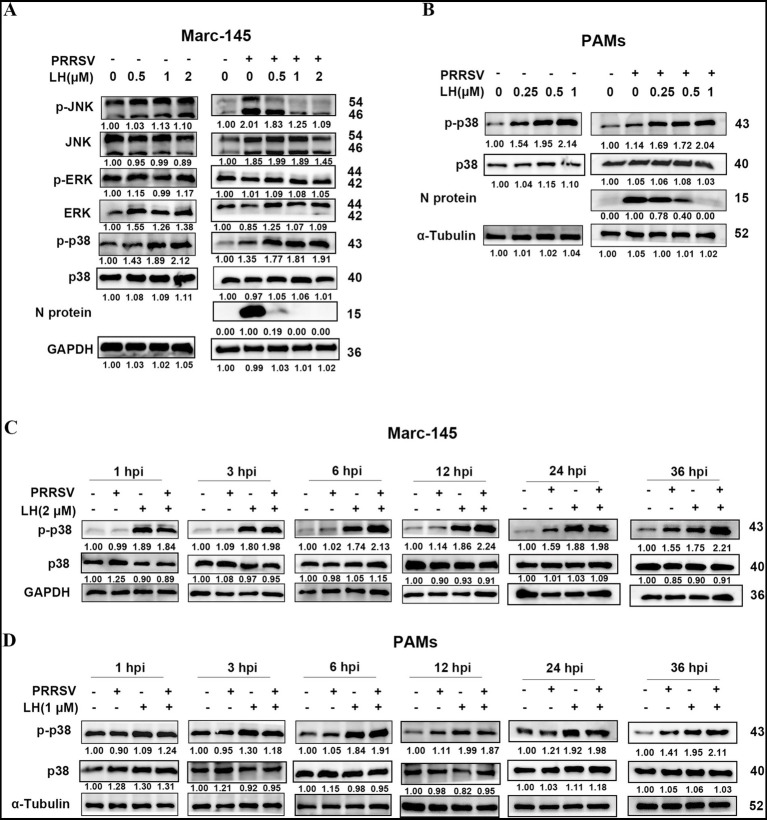
LH upregulates cellular p38 phosphorylation at the early phase in Marc-145 cells and PAMs. **(A)** Marc-145 cells grown in 12-well plates were infected or mock-infected with PRRSV GD-HD (100 TCID_50_) for 2 h at 37°C, followed by incubation in fresh medium containing different concentrations of LH (0.5, 1 or 2 μM). **(B)** PAMs were cultured in 12-well plates and then infected or mock-infected with PRRSV GD-HD (1,000 TCID_50_) for 2 h at 37°C, followed by incubation in fresh medium containing different concentrations of LH (0.25, 0.5 or 1 μM). The cells were collected at 24 hpi and total protein was extracted from cell lysates. The expression levels of MAPK proteins were analyzed by Western blotting **(A,B)**. Marc-145 cells **(C)** or PAMs **(D)** were grown in 12-well plates, cells were infected or mock-infected with PRRSV GD-HD (100 TCID_50_ or 1,000 TCID_50_ for Marc-145 cells and PAMs, respectively) for 2 h at 37°C. Subsequently, the cells were cultured in fresh medium containing 2 μM (for Marc-145 cells) or 1 μM (for PAMs) of LH. The cells were collected at 1, 3, 6, 12, 24 or 36 hpi, and total protein was extracted from cell lysates. The expression levels of p38 and p-p38 were analyzed by Western blotting.

Since LH inhibited PRRSV replication in pre-treatment mode, we speculated that LH may inhibit PRRSV infection by affecting host factors. Lycorine was reported to exhibit cytotoxic effects on human osteosarcoma cell-lines SJSA-1 and U2OS via inducing G1 phase cell cycle arrest and cellular apoptosis by activating p38 MAPK and p53-dependent apoptotic program (Ning et al., 2020). In addition, previous studies have demonstrated that MAPKs can play an important role in host response to virus infections (Arthur and Ley, 2013). Thus, we investigated the potential impact of LH on MAPK signaling pathways by evaluating the effect of LH on expressions of p-JNK, p-ERK and p-p38 in Marc-145 cells. As shown in Figure 5A, PRRSV infection promoted phosphorylation of JNK, ERK and p38 in Marc-145 cells, reflected by increased levels of p-JNK, p-ERK and p-p38. Intriguingly, LH treatment reduced the expression of p-JNK caused by PRRSV infection, while it did not significantly change p-JNK level in PRRSV mock-infected Marc-145 cells (Figure 5A). Impressively, LH treatment significantly increased p-p38 levels in a dose-dependent manner in both PRRSV infected and uninfected Marc-145 cells (Figure 5A) and PAMs (Figure 5B), compared to their controls. These results demonstrate that LH could up-regulate cellular p-p38 level, which might be associated with its inhibition on PRRSV replication.

It was known that PRRSV inhibits the activation of host antiviral innate immune responses, especially during the earlier stages of virus infection (Beura et al., 2010). To clarify the effects of PRRSV infection and LH treatment on the activation of p38, we investigated dynamic effects of LH treatment on p-p38 expression in PRRSV-infected or uninfected Marc-145 cells and PAMs from 1 hpi to 36 hpi. As shown in Figure 5C, PRRSV infection did not induce increased p-p38 expression in Marc-145 cells at and before 12 hpi. However, at 24 and 36 hpi, p-p38 levels significantly increased in PRRSV infected Marc-145 cells. In PAMs, PRRSV infection induced increased p-p38 level only at 36 hpi rather than before this time point (Figure 5D). These results indicated that PRRSV infection induced a late p38 up-regulation in PAMs and Marc-145 cells. This peculiarity was also observed in Marc-145 cells infected with 10 times higher PRRSV dose (1,000 TCID_50_), in which such a higher dose PRRSV did not induce an obvious p-p38 up-regulation before 12 hpi (Supplementary Figure S3). On the contrary, LH induced a rapid boosting of p-p38, reflected by obviously increased p-p38 level at 1 hpi in PRRSV infected and uninfected Marc-145 cells (Figure 5C) and at 3 hpi in PRRSV infected and uninfected PAMs (Figure 5D), and the increased p-p38 levels lasted to 36 hpi compared to the LH-absent controls (Figures 5C,D). These results collectively demonstrated that LH could induce a rapid boosting of p38 MAPK while PRRSV infection induced a late up-regulation of this pathway in cell cultures.

### P-p38 up-regulation attenuates PRRSV replication

**Figure 6:**
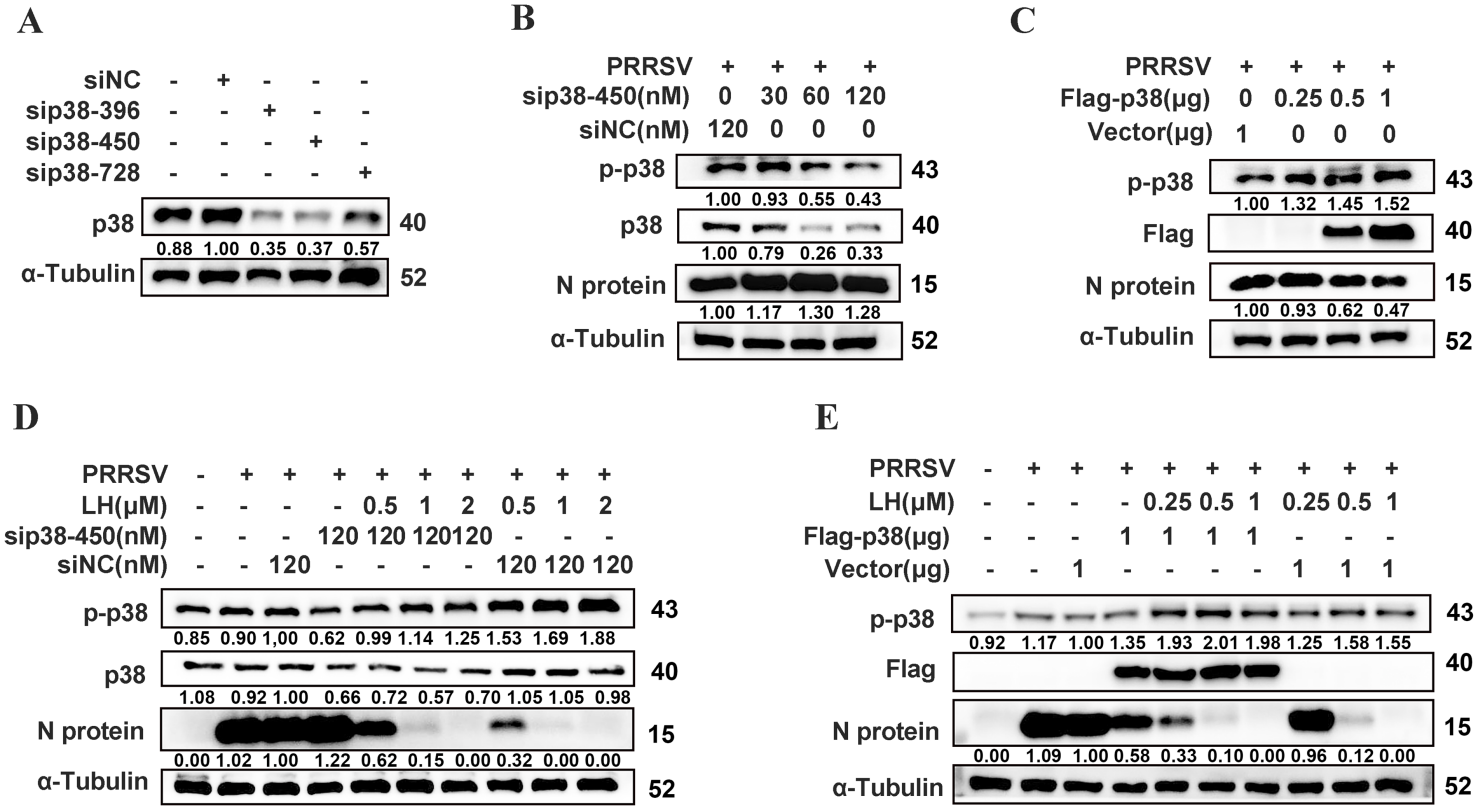
The up-regulation of p-p38 suppresses PRRSV replication in Marc-145 cells. **(A)** The knockdown efficiencies of three p38 specific siRNAs or control siRNA (NC) against p38 were analyzed by Western blotting at 24 h after transfection. **(B)** Marc-145 cells were transfected with sip38-450 (30, 60 or 120 nM) or siNC for 24 h and then infected with PRRSV GD-HD (100 TCID_50_) for 2 h. The indicated proteins were quantified at 24 hpi by Western blotting. **(C)** Marc-145 cells were transfected with Flag-tagged p38 (0.25, 0.5 or 1 μg) or empty vector for 24 h and then infected with PRRSV GD-HD (100 TCID_50_) for 2 h. The indicated proteins were quantified at 24 hpi by Western blotting. sip38-450 **(D)** or Flag-tagged p38 **(E)** transfected Marc-145 cells were infected with PRRSV GD-HD (100 TCID_50_) for 2 h and treated with different concentrations of LH (0.5, 1 or 2 μM) for 24 h. The indicated proteins were quantified by Western blotting.

To further investigated the link between p38 activity and PRRSV replication, we explored the effect of siRNA-mediated knockdown of p38 on PRRSV replication. First, we analyzed the knockdown efficiency of p38-specific siRNA (sip38) by Western blotting. To ensure siRNA targeting efficiency, we used three different siRNAs targeting p38 (sip38-396, sip38-450 and sip38-728). Among these siRNAs, sip38-396 and sip38-450 could significantly reduce p38 expression (Figure 6A), and p38 knockdown with sip38-450 led to substantially enhanced N protein expression compared with the siNC control group (Figure 6B), indicating that cellular p-p38 up-regulation has a negative effect on PRRSV replication. We next evaluated the effect of p38 overexpression mediated by plasmid transfection on PRRSV replication. As shown in Figure 6C, p38 overexpression significantly inhibited PRRSV replication in a dose-dependent manner. To confirm that the anti-PRRSV activity of LH is indeed through boosting p-p38 level, Marc-145 cells were transfected with sip38-450 or Flag-p38 for 24 h, then the cells were infected with PRRSV for 2 h followed by treatment with different concentrations of LH (0.5, 1 and 2 μM) for 24 h. The results showed that knockdown of cellular p38 partly reversed PRRSV replication inhibition mediated by LH treatment in Marc-145 cells (Figure 6D). In contrast, p38 overexpression enhanced LH-induced PRRSV inhibition in Marc-145 cells (Figure 6E). Of note, anti-PRRSV activity of LH at 1 and 2 μM was significantly greater than that mediated by p38 overexpression via plasmid transfection (Figures 6C,E). These results collectively demonstrated that p-p38 up-regulation attenuates PRRSV replication and antiviral activity of LH is closely associated with its induction on the activation of p38 MAPK.

### LH enhances Caspase-1-mediated maturation of IL-1β to inhibit PRRSV replication via p38/NLRP3 pathway in Marc-145 cells

**Figure 7:**
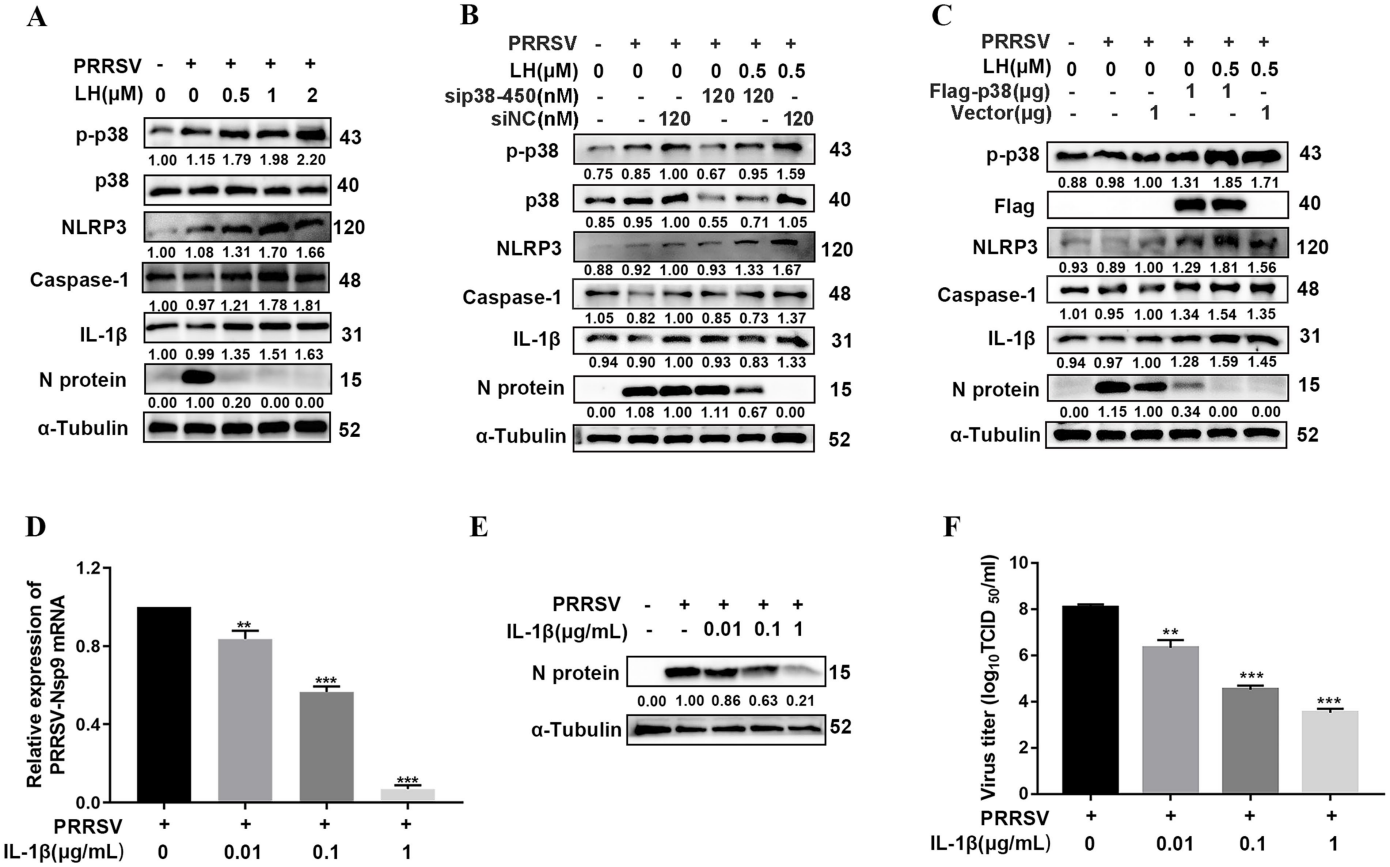
LH promotes IL-1β production to inhibit PRRSV replication in Marc-145 cells. **(A)** Marc-145 cells grown in 12-well plates were infected with PRRSV GD-HD (100 TCID_50_) for 2 h at 37°C, followed by incubation in fresh medium containing different concentrations of LH (0.5, 1 or 2 μM). The cells were collected at 24 hpi. Total protein was extracted from cell lysates and analyzed by Western blotting. **(B,C)** Marc-145 cells were transfected with siNC or sip38-450 **(B)** and Flag-tagged p38 or empty vector **(C)**. After 24 h, the cells were infected with PRRSV GD-HD (100 TCID_50_) and then were treated with LH (0.5 μM). The indicated proteins were analyzed at 24 hpi by Western blotting. **(D–F)** Marc-145 cells grown in 12-well plates were infected with PRRSV GD-HD (100 TCID_50_) for 2 h at 37°C and then cultured in fresh medium containing recombinant porcine interleukin-1β (IL-1β) (0.01, 0.1, or 1 μg/mL). After 24 h, the samples were subjected to qRT-PCR **(D)**, Western blotting **(E)** and viral titer titration **(F)** analyses. Statistical significances are denoted by *p < 0.05, **p < 0.01, and ***p < 0.001, compared with the DMSO control.

The p38 protein is the most important member of the MAPK family controlling the inflammatory response (Ono and Han, 2000). Our previous study revealed that inflammatory mediator IL-1β exhibits inhibition on PRRSV replication in PAMs and Marc-145 cells (Zhang et al., 2022). p38 has been reported as an upstream kinase to regulate the activation of the NLRP3/Caspase-1/IL-1β pathway in an acute lung injure model (Li et al., 2018). To explore whether LH inhibits PRRSV infection via inducing p38-dependent IL-1β expression, we investigated the effects of LH on the expressions of IL-1β and related inflammatory proteins at 24 hpi by Western blotting. As shown in Figure 7A, LH treatment induced significantly increased expressions of NLRP3, Caspase-1 and IL-1β in a dose-dependent manner in PRRSV infected Marc-145 cells, while PRRSV infection did not affected the expressions of the three inflammatory factors. These results indicated that anti-PRRSV activity of LH might be associated with its activation on p38-dependent NLRP3/Caspase-1/IL-1β pathway. Meanwhile, LH treatment did not significantly affect the expressions of p-p65 and HMGB1 proteins (Supplementary Figure S4), suggesting that LH has negligible impact on the NF-κB signaling pathway.

NLRP3 inflammasome plays a key role in defense against microbial infections (Kelley et al., 2019), and p38 has been reported as an upstream kinase to regulate NLRP3 inflammasome activation (Dong et al., 2021). To further clarify whether LH exerts antiviral effect by activating the NLRP3/Caspase-1/IL-1β pathway through inducing p38 phosphorylation, we examined the effects of LH treatment on the NLRP3/Caspase-1/IL-1β pathway in p38 knockdown or overexpressed PRRSV-infected Marc-145 cells. Results in Figure 7B showed that p38 silencing partly reversed LH induced up-regulations of p-p38, NLRP3, Caspase-1 and IL-1β levels. On the contrary, overexpression of p38 enhanced the expressions of p-p38, NLRP3, Caspase-1 and IL-1β proteins, and it also promoted LH-induced productions of the above inflammatory proteins (Figure 7C). These results collectively affirmed that LH activates the NLRP3/Caspase-1/IL-1β pathway through inducing p38 phosphorylation and thus exerts antiviral effects.

IL-1β, mainly produced by monocytes and macrophages, is an essential pro-inflammatory cytokine involved in host innate immune responses against pathogenic microbial infections (Yazdi and Ghoreschi, 2016). Our previous study revealed that inflammatory mediator IL-1β exhibits inhibition on PRRSV replication in PAMs and Marc-145 cells (Zhang et al., 2022). In this study, the antiviral effect of IL-1β against PRRSV infection in Marc-145 cells was confirmed. As shown in Figures 7D–F, treatment of exogenous recombinant IL-1β (0.01, 0.1 and 1 μg/mL) on PRRSV infected Marc-145 cells resulted in significant decreases of the expressions of viral mRNA, N protein and virus titer in a dose-dependent manner, while these IL-1β concentrations did not affect Marc-145 cell’s viability (Supplementary Figure S5A).

### LH inhibits PRRSV replication in PAMs via up-regulating the p38 MAPK/NLRP3/Caspase-1/IL-1β pathway

**Figure 8:**
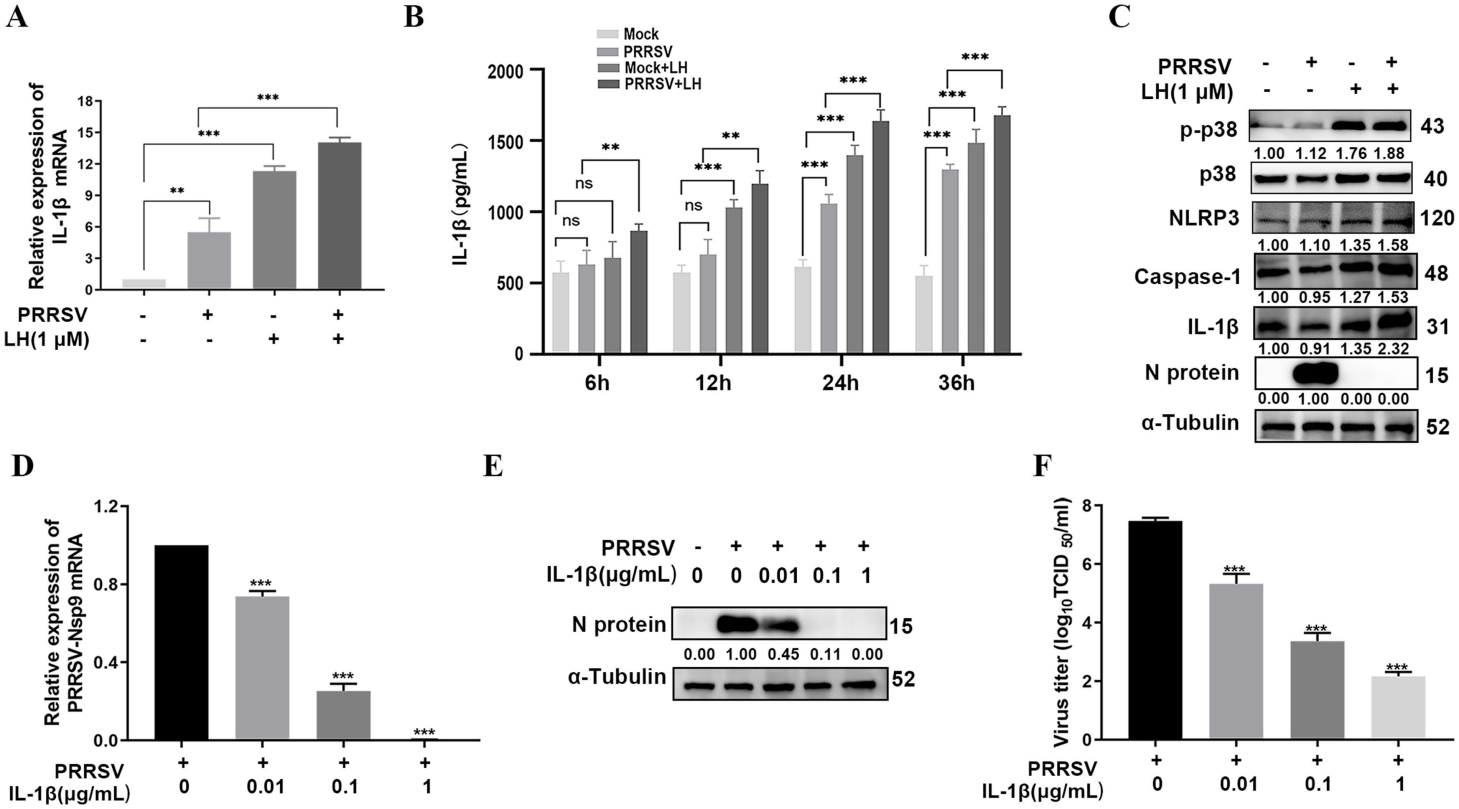
LH induces up-regulation of the p38 MAPK/NLRP3/Caspase-1/IL-1β pathway to inhibit PRRSV replication in PAMs. PAMs cultured in 12-well plates were infected or uninfected with PRRSV (1,000 TCID_50_) for 2 h at 37°C. Subsequently, the cells were cultured in fresh medium containing 1 μM LH. After 24 h, the cells were harvested for qRT-PCR analyses to assess the mRNA level of IL-1β **(A)**. The cells supernatants were harvested at 6, 12, 24 and 36 h post LH treatment for detecting IL-1β levels using ELISA **(B)**. Total protein was extracted from cell lysates at 24 hpi, and the levels of indicated proteins in the cells were analyzed by Western blotting **(C)**. **(D–F)** Cells cultured in 24-well plates were infected with PRRSV GD-HD (1,000 TCID_50_) for 2 h at 37°C and then incubated in fresh medium containing various concentrations of IL-1β. At 24 hpi, the samples were subjected to qRT-PCR **(D)**, Western blotting **(E)** and viral titer titration **(F)**. Statistical significances are denoted by *p < 0.05, **p < 0.01, and ***p < 0.001. ns: no significant difference, compared with the DMSO control.

Given that PAMs are natural target cells in pigs, we examined if LH could enhance Caspase-1-mediated IL-1β production by promoting activation of p38/NLRP3, which in turn suppresses PRRSV replication in PAMs. Results in Figure 8A showed that LH treatment at 1 μM for 24 h significantly increased mRNA expressions of IL-1β in both PRRSV infected and uninfected cells. In addition, LH treatment led to ongoing increase of the IL-1β level in both PRRSV infected and uninfected PAMs from 6 hpi to 36 hpi (Figure 8B). Meanwhile, LH treatment for 24 h led to significant increases of p-p38, NLRP3 and Caspase-1 levels in PAMs (Figure 8C), in line with its effects in Marc-145 cells (Figures 6, 7). Moreover, the addition of exogenous recombinant IL-1*β* at non-cytotoxic concentrations (0.01, 0.1, and 1 μg/mL) elicited a similar inhibitory profile on PRRSV replication in PAMs (Figures 8D–F), mimicking its effects in Marc-145 cells. These results collectively indicated that LH inhibits PRRSV replication in PAMs through up-regulating the p38 MAPK/NLRP3/Caspase-1/IL-1β pathway.

## Discussion

PRRSV remains a significant threat to the swine industry worldwide due to the lack of effective measures for preventing and treating this disease. Therefore, there is an urgent need to develop novel antiviral strategies to combat ongoing PRRSV pandemics. Previous studies have shown that LH possesses a wide range of biological activities, such as antitumoral, antiviral and antibacterial effects (Cao et al., 2013). In this study, we revealed for the first time that LH potently inhibits PRRSV replication in Marc-145 cells nd PAMs with EC_50_ values ranging from 0.43 to 0.67 μM, which are far below the detectable cytotoxicity threshold. Mechanistically, we demonstrated that LH induces a rapid up-regulation of p-p38, leading to increased expression of NLRP3 and Caspase-1, which in turn promotes the secretion of IL-1β, thereby suppressing PRRSV propagation.

In viral infections, activation of cell signaling pathways is part of the cellular defense mechanism with the basic aim of inducing an antiviral state. However, viruses can exploit enhanced cell signaling activities to support various stages of their replication cycles. p38 MAPK plays an important role in the signaling cascade induced by various extracellular stimuli, including viral infections. Depending on the nature of the virus, p38 MAPK signaling may either support or inhibit viral replication. For instance, p38 MAPK is needed for efficient synthesis of SARS-CoV-2 subgenomic mRNA (Higgins et al., 2023). p38 MAPK is also known to support replication of multiple other viral families, including IAV, respiratory syncytial virus (RSV), HBV, herpes simplex virus-1 (HSV-1), coxsackievirus, Newcastle disease virus (NDV), rotavirus, encephalomyocarditis virus, and enterovirus (Chander et al., 2021). Pharmaceutically p38 MAPK inhibition leads to decreased virus replication; for example, NJK14047 blocks synthesis of HBV RNA (Kim et al., 2017) and BX-795 inhibits p38-mediated expression of HSV-1 genes (Su et al., 2017). Whereas, in some virus infections, the activation of p38 MAPK inhibits viral replication through various mechanisms, including the promotion of inflammatory responses and the induction of antiviral gene expression (Chander et al., 2021). For instance, in porcine deltacoronavirus (PDCoV) infection, the activation of p38 MAPK-C/EBP-β pathway elicits inflammatory responses and promotes the expression of complement component 3, which suppresses PDCoV replication (Chen et al., 2024). Andrographolide, a plant-derived diterpene lactone, clinically used as anti-inflammatory drug in China, was reported to suppress hepatitis C virus (HCV) replication by promoting the antiviral IFN responses via activating p38 phosphorylation and thereby stimulating nuclear factor erythroid 2-related factor 2 (Nrf2)–mediated HO-1 expression (Lee et al., 2014). Another two compounds, Rupestonic acid derivative YZH-106 (Ma et al., 2016) and 6-demethoxy-4’-O-methylcapillarisin (Zhong et al., 2019), were reported to impair IAV replication by similar antiviral mechanism as andrographolide against HCV. These studies indicated that the effects of p38 MAPK signaling on various virus replications are quite complicated and even inconsistent in different reports for the same virus infection (such as IAV).

Quite a few previous studies have demonstrated that PRRSV is able to suppress host immune responses to benefit itself replication, including poor interferon alpha (IFN-*α*) production, delayed and weak neutralizing antibody response, and lower T cell mediated immune response (Lowe et al., 2005). However, little is known about dynamic change of p38 MAPK signaling during PRRSV infection. Hou et al. showed that PRRSV CH-1a infection induced p38 phosphorylation in bone marrow-derived macrophages (BMDMs) at 12 and 24 hpi, and the p-p38 level at 36 hpi is dramatically decreased. Notably, p38 was not activated by the virus infection before 12 hpi (Hou et al., 2012). Similar dynamic change of p-p38 production was also reported by Lee in a PRRSV VR2332 infection in immortalized PAM cells (Lee and Lee, 2012). Results from the two studies indicates that PRRSV infection induces a late rather than an early activation of p38 MAPK signaling. In our study, PRRSV GD-HD infection induces late activations of p38 MAPK at 24 hpi in both Marc-145 cells and PAMs (Figures 5C,D), which is consistent with the above previous studies. Regarding to the effect of pharmaceutical inhibition on PRRSV replication, Hou et al. reported that pre-treatment of BMDMs with a selective p38 inhibitor SB203580 before virus infection did not affect PRRSV replication (Hou et al., 2012), while Lee showed that treatment with another selective p38 inhibitor SB202190 on PAM-pCD163 cells throughout infection impairs PRRSV replication (Lee and Lee, 2016). Here, we showed for the first time that LH treatment induces a rapid up-regulation of p-p38 level (at 1 h post LH addition) in both PRRSV-infected and mock-infected Marc-145 cells and PAMs (Figures 5C,D). Interestingly, LH treatment did not affect expressions of p-JNK and p-ERK in PRRSV-mock infected Marc-145 cells (Figure 5A). Furthermore, we revealed that early boosting of p38 phosphorylation by LH treatment leads to markedly inhibition of PRRSV replication (Figures 5A,B, 6D,E). Results in Figures 6B–E showed that p38 silencing promotes PRRSV replication, while p38 overexpression suppresses the virus replication; and p38 silencing reversed the antiviral effects of LH, while p38 overexpression enhanced the antiviral effects of LH (Figures 6D–E). These data further confirmed that early boosting of p38 phosphorylation is detrimental for PRRSV replication and LH exerts its anti-PRRSV activity by promoting p38 MAPK signaling at an early stage of the virus infection. However, it cannot be completely ruled out that other mechanisms may participate in LH’s anti-PRRSV effects, requiring further investigation. The activation of p38 MAPK is driven by a variety of extracellular and intracellular signals, which include stress signals, pro-inflammatory cytokines, and pathogen recognition. The key upstream kinases responsible for activating p38 include TAK1, ASK1, and MKK3/6, which act as intermediaries between external stimuli and p38 activation (Wilson et al., 1996; Matsukawa et al., 2004; Sato et al., 2005). Cytokine signaling, particularly through receptors like TNF receptor 1 (TNFR1) and IL-1 receptor, triggers the activation of these kinases via pathways involving adaptor proteins such as MyD88 and TRAF6, leading to the phosphorylation of MKK3/6 and subsequent activation of p38 (Sabio and Davis, 2014). Oxidative stress, induced by reactive oxygen species (ROS), is another critical factor that activates p38 MAPK, especially during cellular stress and inflammation. This activation often occurs via ASK1, a kinase that is sensitive to oxidative signals and directly phosphorylates p38 (Dolado et al., 2007). Furthermore, viral infections also activate p38 through pattern recognition receptors (PRRs) such as TLRs and RIG-I-like receptors, which detect pathogen-associated molecular patterns (PAMPs) (Kawai and Akira, 2010). While significant progresses have been made in understanding how various stimuli activate p38 MAPK, the mechanism by which LH induces p38 activation in Marc-145 cells and PAMs is still to be revealed.

IL-1β is a key pro-inflammatory cytokine and plays a very important role in shaping the inflammatory response against pathogens. The level of IL-1β is strongly associated with the development of many diseases. An appropriate amount of IL-1β can repair damage and reduce viral proliferation, whereas excessive IL-1β can increase morbidity and mortality. Previous studies have demonstrated that many viruses, including IAV, infectious bronchitis virus (IBV), and NDV induced the production of IL-1β in infected cells via different signaling pathway (Thomas et al., 2009; Amarasinghe et al., 2018; Gao et al., 2020). In addition, IL-1β has been demonstrated to suppress replication of some viruses, including HCV (Guo et al., 2020), ASFV (Li J. et al., 2021) and HBV (Li et al., 2022). NLRP3 inflammasome, a multiprotein complex, is known as an essential mediator to activate Caspase-1 which initiates the processing and release of pro-inflammatory cytokines IL-1β and IL-18 (Blevins et al., 2022). The p38 MAPK signaling has a dual function in the activation and expression of NLRP3 inflammasome. On the one hand, p38 phosphorylation increases the formation of the multi-protein complex of NLRP3 and pro-Caspase-1, and enhances the functional role of NLRP3 inflammasome; on the other hand, it has been reported that inhibition or depletion of p38 MAPK can lead to over-activation of NLRP3 inflammasome in the activation phase, exerting certain anti-inflammatory effects (Wang et al., 2024). In our study, PRRSV infection led to an increase in IL-1β secretion in PAMs at the late infection stage (24 hpi) (Figure 8B), in lines with the time point of p38 MAPK signaling activation (Figures 5C,D), confirming that PRRSV infection induces a delayed antiviral inflammatory response. More importantly, LH treatment induced a rapid up-regulation of IL-1β production at 6 and 12 hpi in PRRSV-infected PAMs (Figure 8B), and it also promoted the expressions of NLRP3 and Caspase-1 in a dose-dependent manner in PRRSV-infected Marc-145 cells and PAMs (Figures 7, 8). Moreover, p38 silencing reversed these effects, while p38 overexpression amplified them in PRRSV-infected Marc-145 cells (Figures 7B,C). Meanwhile, we observed that exogenous addition of IL-1β could significantly inhibit the proliferation of PRRSV in both Marc-145 cells and PAMs (Figures 7D–F, 8D–F). These results collectively demonstrated that LH enhances Caspase-1-mediated maturation and release of IL-1β to inhibit PRRSV replication in an p38 MAPK pathway-dependent manner. One previous study has shown that IL-1β induces an increase in the expression of the membrane-disintegrating protein ADAM17 (Lanaya et al., 2014), which has been shown to inhibit PRRSV entry by down-regulating the expression of CD163 on the cell membrane (Guo et al., 2014). However, the precise mechanism by which IL-1β exerts its anti-PRRSV effects remains to be further elucidated.

Among diverse pharmacological activities of lycorine and LH, anticancer activities are the most extensively studied in both cellular and animal models (Zhang et al., 2024). Administering 5–30 mg/kg lycorine and LH significantly inhibited tumor growth in the xenograft mouse model, mostly conducted using nude mice and BALB/c mice. For examples, the administration of 20 mg/kg/day of lycorine for 40 days via intraperitoneal injection (i.p.) injection resulted in almost complete tumor suppression in luciferase-expressing U251-bearing nude BALB/c mice (Shen et al., 2018). The administration of 5 mg/kg of lycorine by i.p. injection for 30 days suppressed lung metastasis by about 80% in the tail vein MDA-MB-231-Luc injection model, with less weight loss (Ying et al., 2017). Lycorine and LH were reported to has low toxicity to healthy mice. Following i.p. administration of LH in mice, the LD_50_ was calculated to be 112.2 mg/kg; while administered orally, the LD_50_ increased to 344 mg/kg (Ch'en and Li, 1965). Following single administration of LH by subcutaneous or intravenous injection at 1 mg/kg in Beagle dogs, maximum plasma concentrations (Cmax) reached 2.37 μg/mL (= 8.25 μM) and 5.92 μg/mL (= 20.6 μM), respectively, which are much higher than the anti-PRRSV EC_50_ values of LH in Marc-145 cells and PAMs (ranging from 0.43 to 0.67 μM) (Figures 1, 3). The safety and pharmacokinetics characteristicses of LH in animals from previous studies indicate that anti-PRRSV LH concentrations in pigs are easy to be achieved.

In conclusion, while PRRSV infection induced a delayed activation of p38 MAPK/NLRP3 pathway and production of IL-1β, early boosting of p38 MAPK signaling pathway mediated by LH treatment potently inhibited the virus replication in Marc-145 cells and PAMs via enhancing the production of NLRP3 and its down-stream inflammatory cytokine IL-1β. Early boosting of p38 MAPK pathway may thus represent a potential strategy to control PRRSV infection, positioning LH as a novel therapeutic agent for combating this infectious disease. Future research should prioritize: (1) evaluating LH’s antiviral efficacy in PRRSV-infected pigs alongside comprehensive assessments of its *in vivo* safety profile and pharmacokinetics; (2) conducting chemical modifications to enhance LH’s *in vivo* antiviral activity; and (3) investigating synergistic effects between LH (or its structural analogs) and existing antiviral compounds.

## Data Availability

The data generated during this study are available from the corresponding authors upon reasonable request.
