# Supplementary material for: Early boosting of p38 MAPK signaling pathway by lycorine hydrochloride potently inhibits PRRSV proliferation in primary and established cells
Source: Front Microbiol. 2025 Aug 21;16:1664973. doi: 10.3389/fmicb.2025.1664973 (PMC12408688; doi:10.3389/fmicb.2025.1664973)
Supplement: Supplementary file 1 [file Data_Sheet_1.pdf]

Figure S1 The protein expression levels were detected by Western blot (corresponding to Figure 2 B)

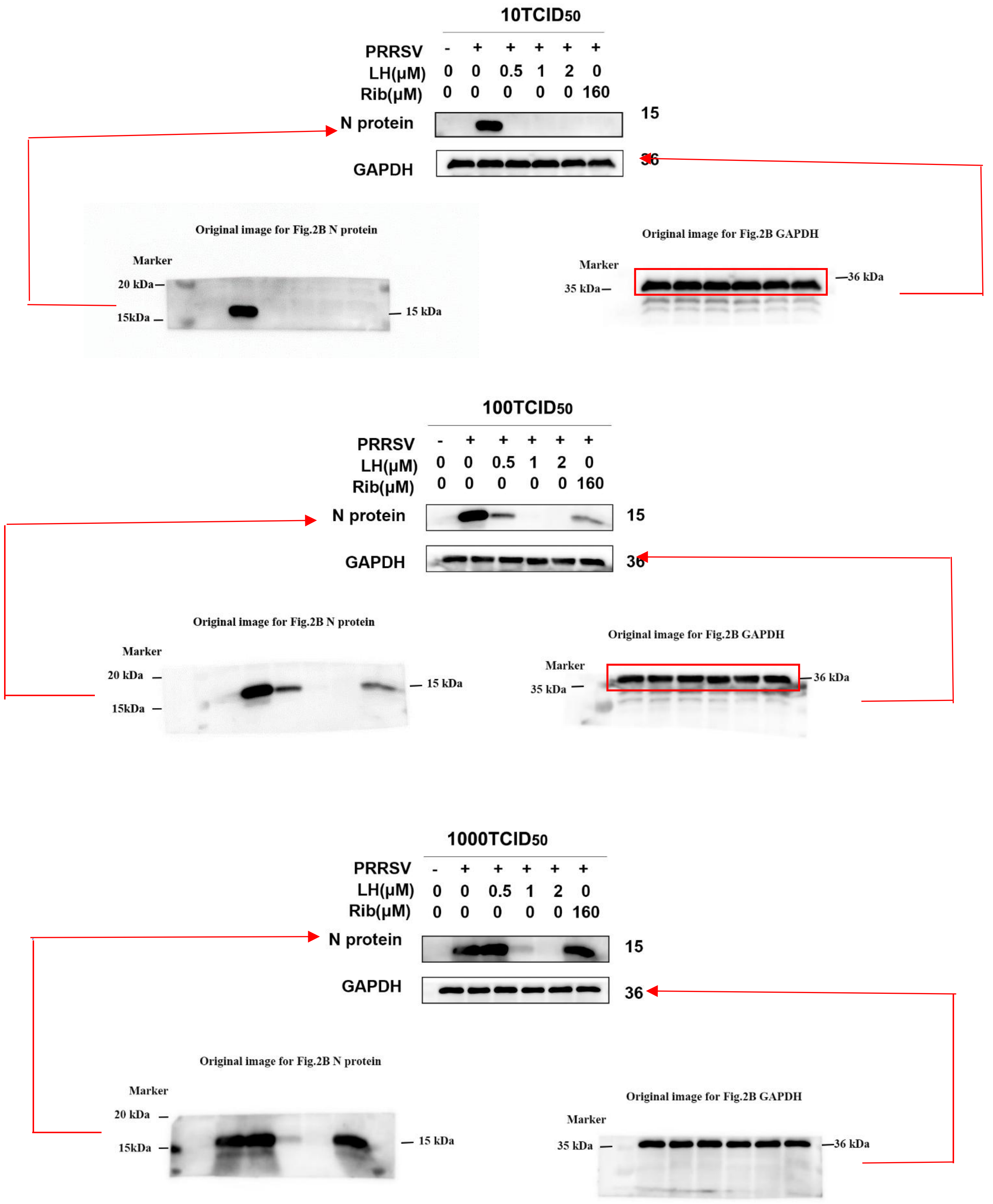

Figure S2 The protein expression levels were detected by Western blot (corresponding to Figure 2E)

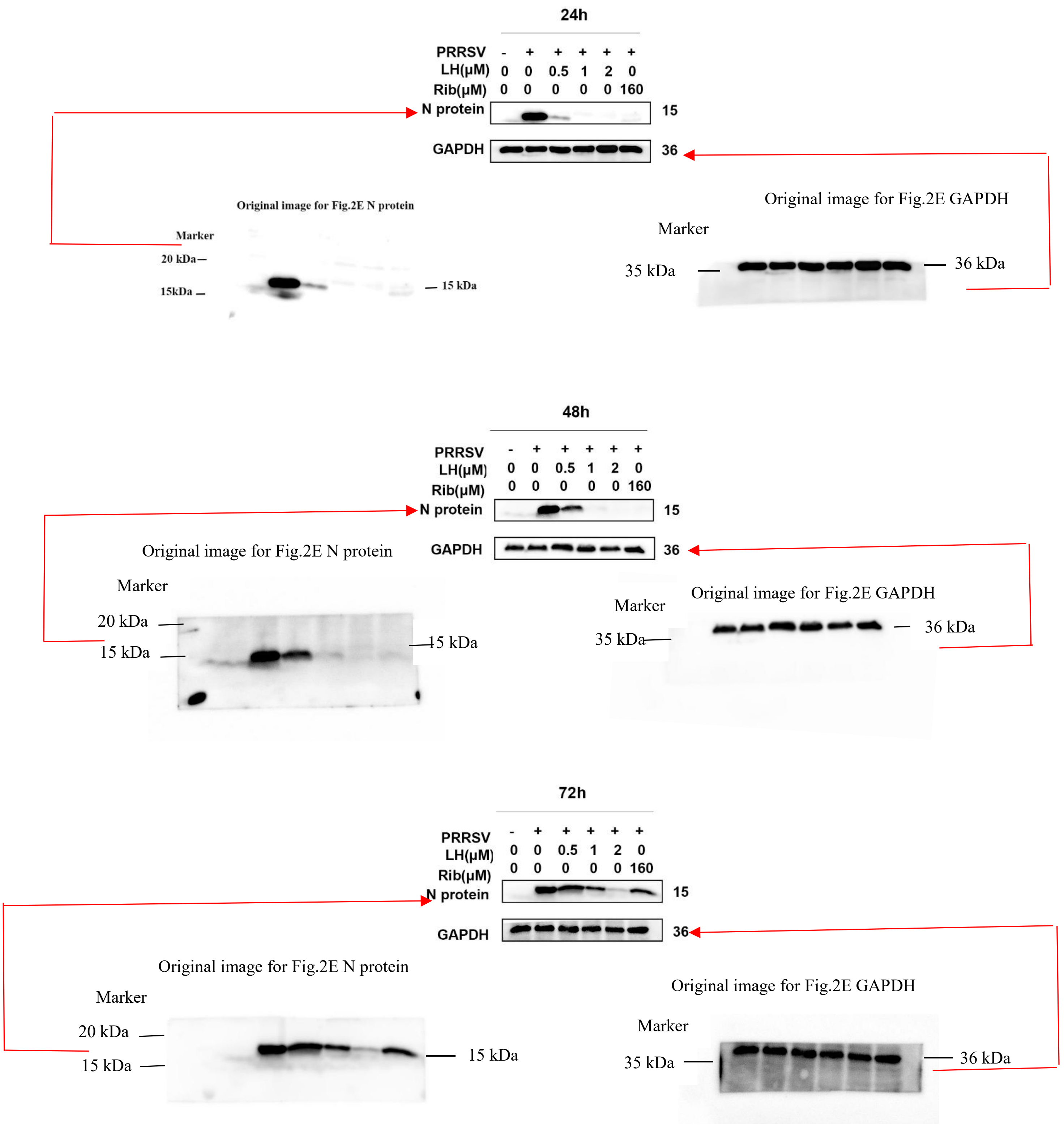

Figure S3 The protein expression levels were detected by Western blot (corresponding to Figure 5A)

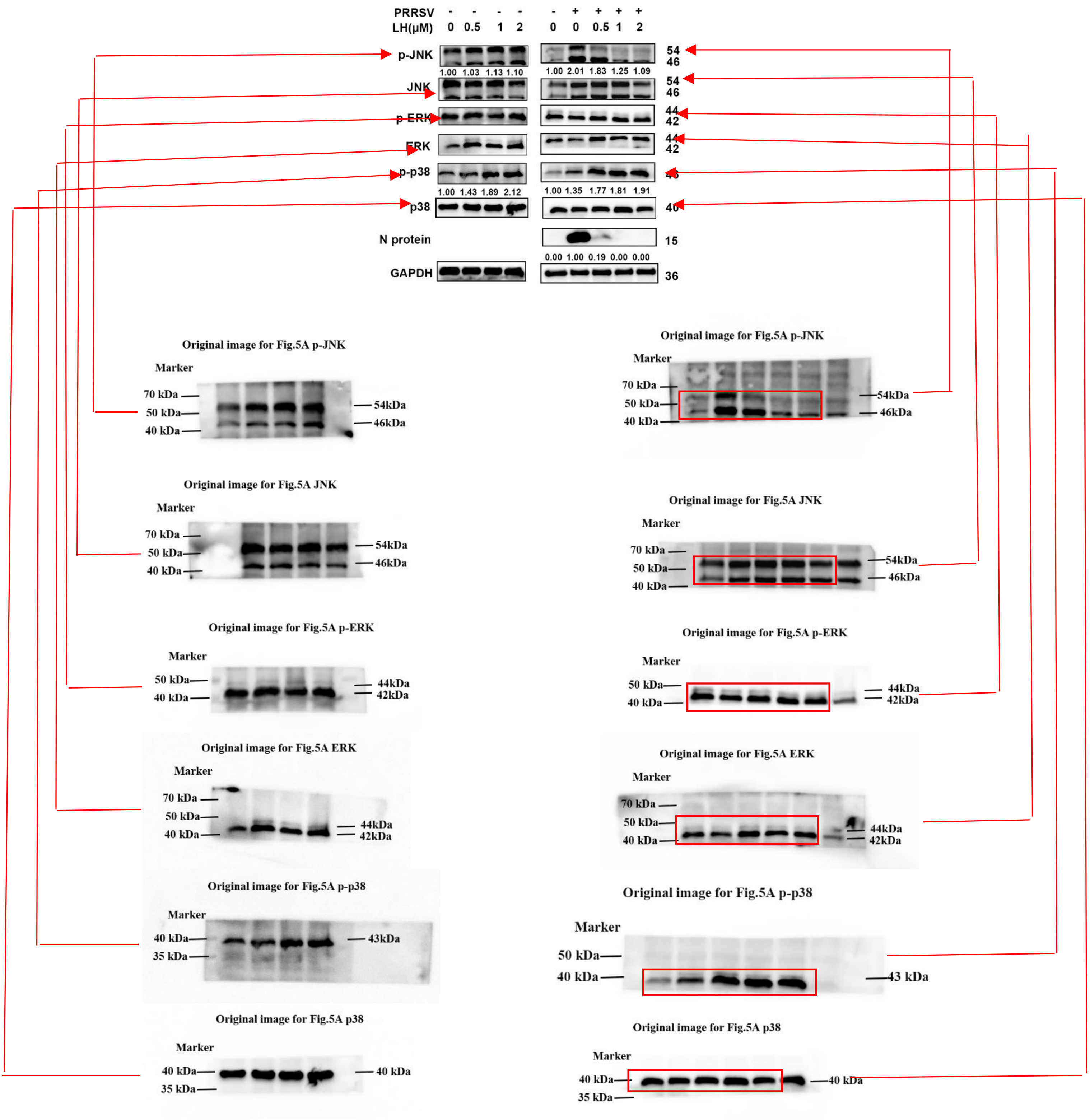

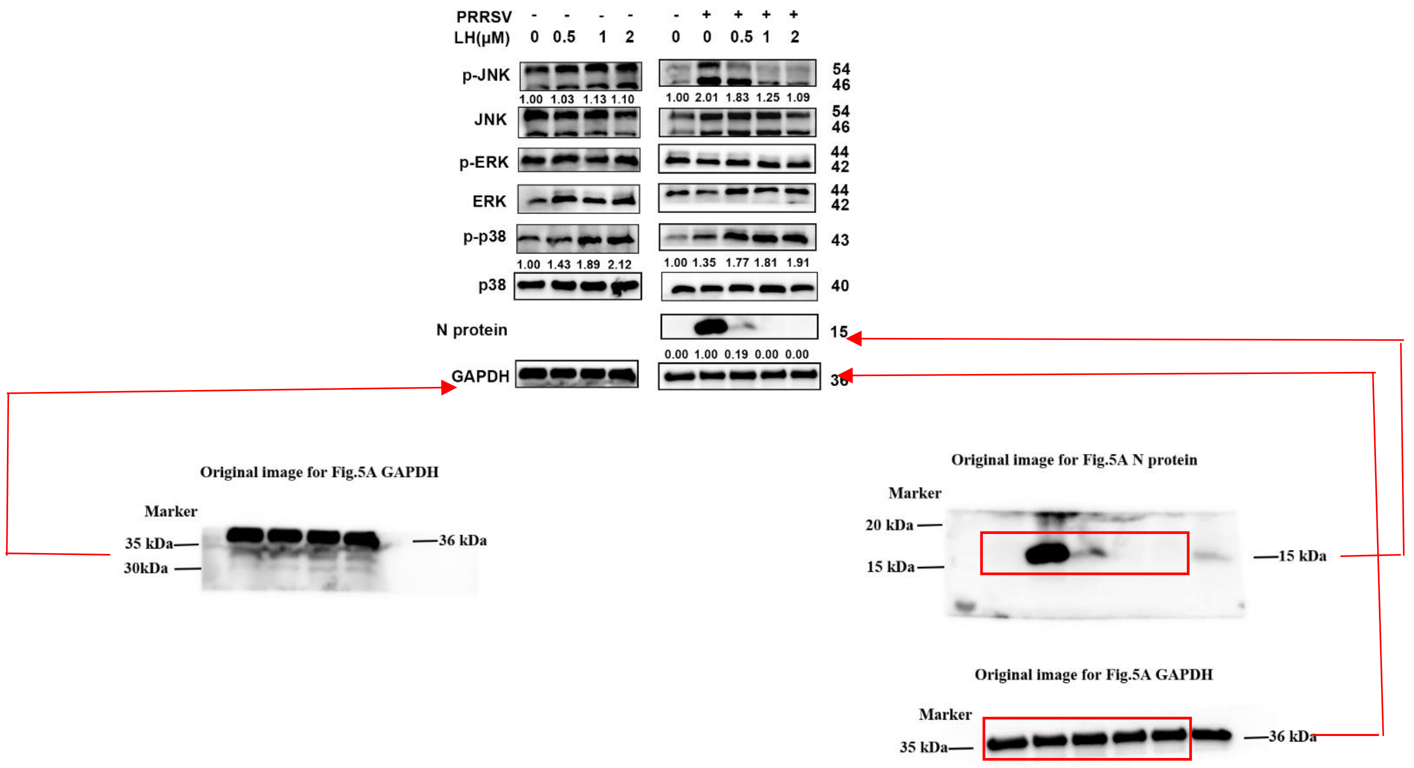
PRRSV - - - - - + + + +
LH(μM) 0 0.5 1 2 0 0 0.5 1 2
p-JNK
1.00 1.03 1.13 1.10
1.00 2.01 1.83 1.25 1.09
54
46
JNK
54
46
p-ERK
44
42
ERK
44
42
p-p38
43
1.00 1.43 1.89 2.12
1.00 1.35 1.77 1.81 1.91
p38
40
N protein
15
0.00 1.00 0.19 0.00 0.00
GAPDH
36
Original image for Fig.5A GAPDH
Marker
35 kDa
30kDa
36 kDa
Original image for Fig.5A N protein
Marker
20 kDa
15 kDa
15 kDa
Original image for Fig.5A GAPDH
Marker
35 kDa
36 kDa

Figure S4 The protein expression levels were detected by Western blot (corresponding to Figure 5B)

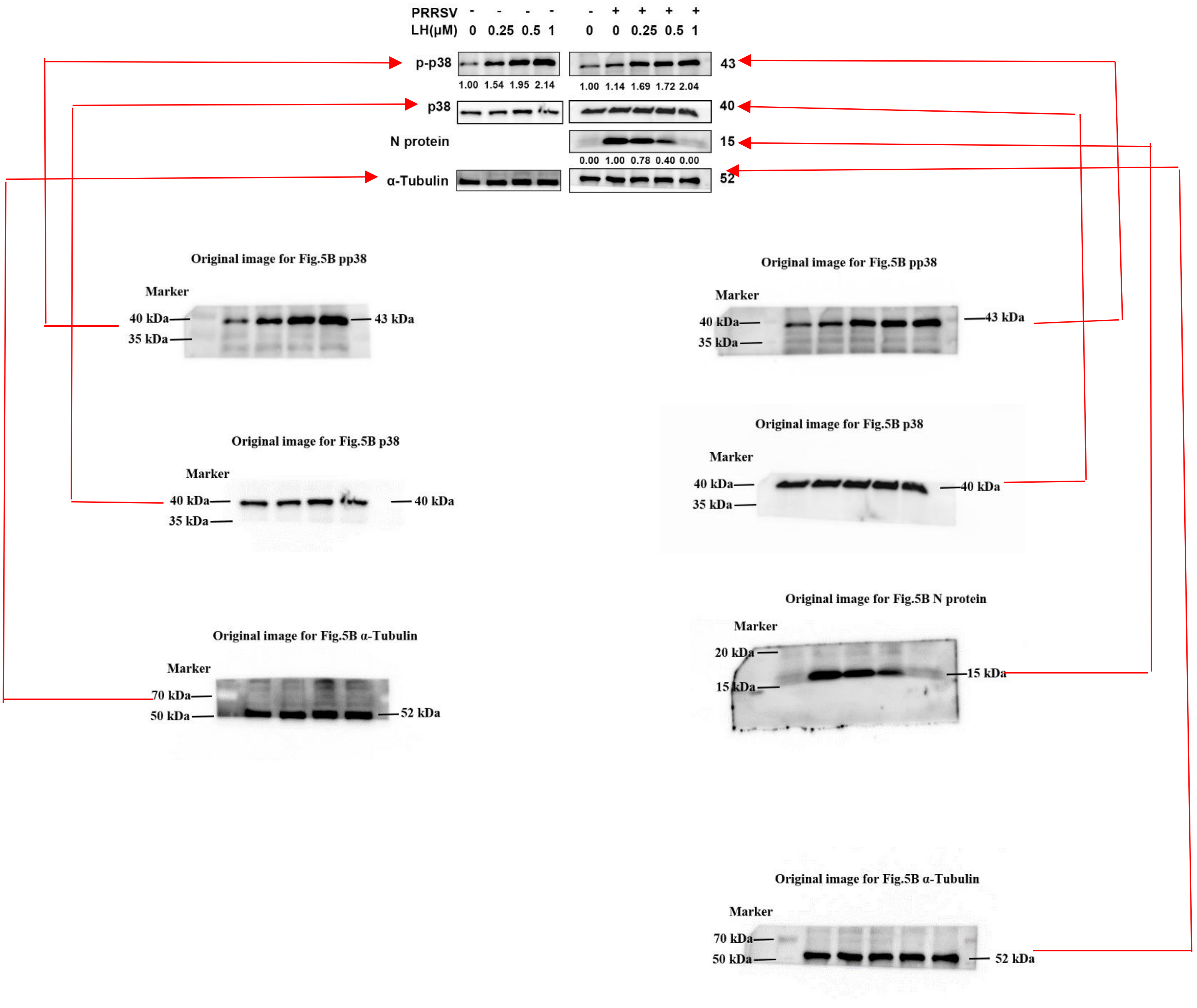

Figure S5 The protein expression levels were detected by Western blot (corresponding to Figure 5C)

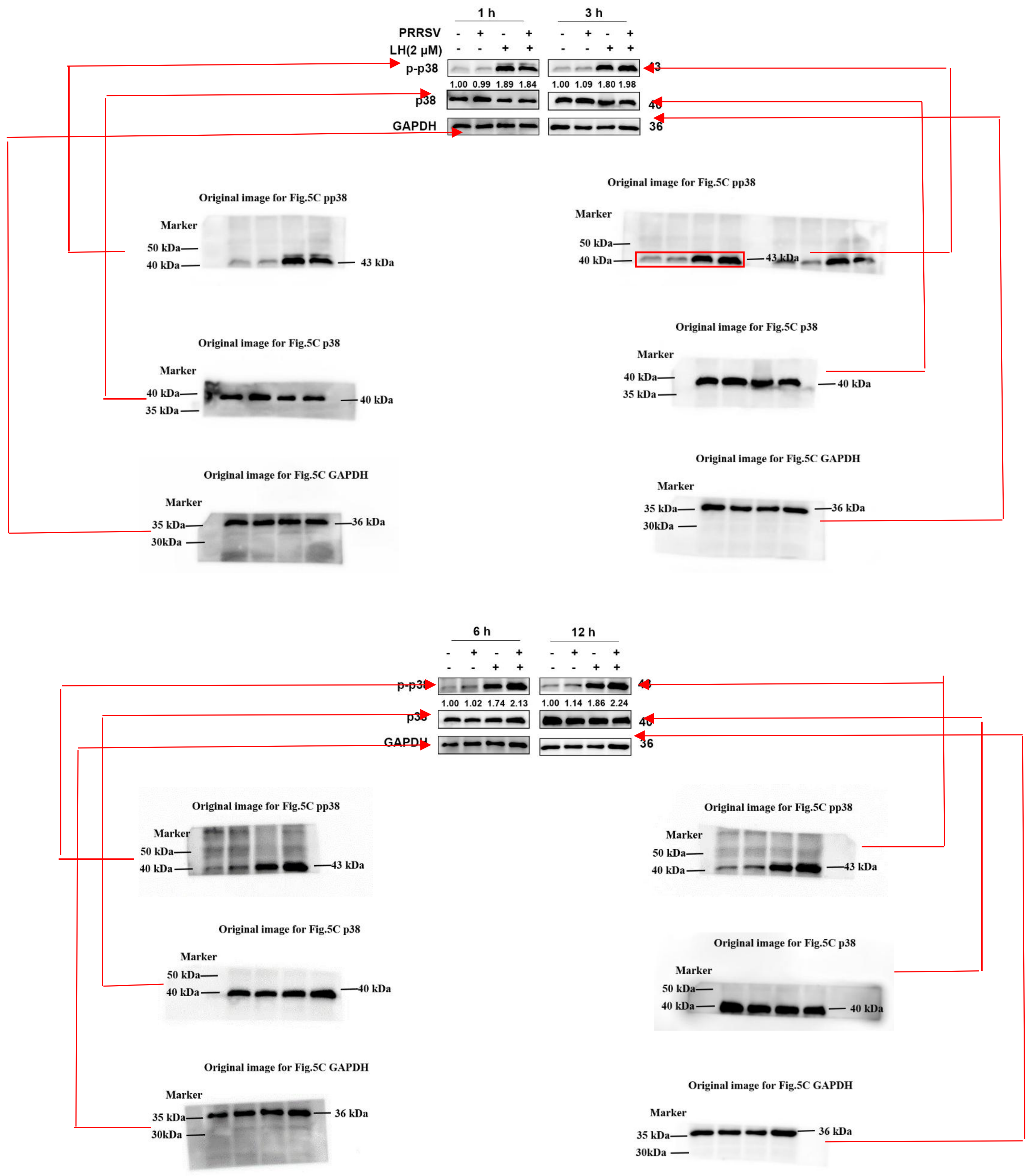

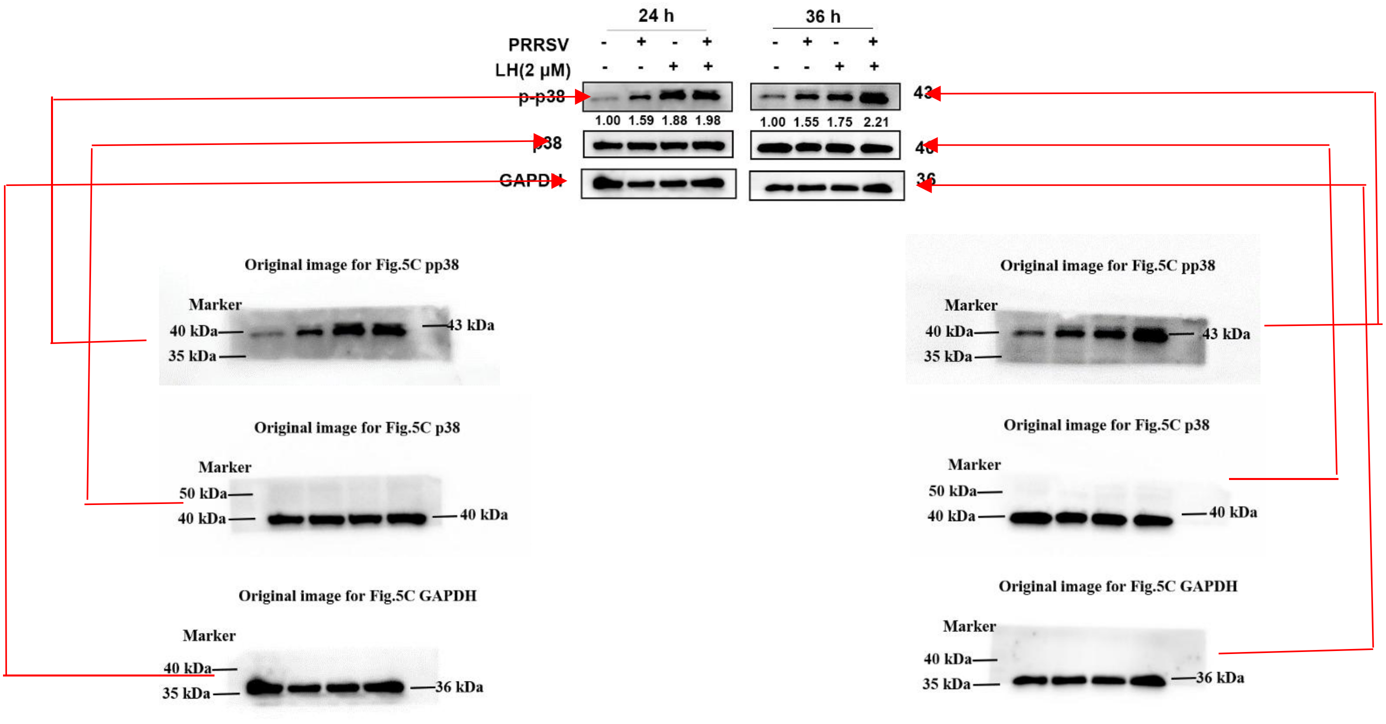

Figure S6 The protein expression levels of N protein and GAPDH were detected by Western blot (corresponding to Figure 5D)

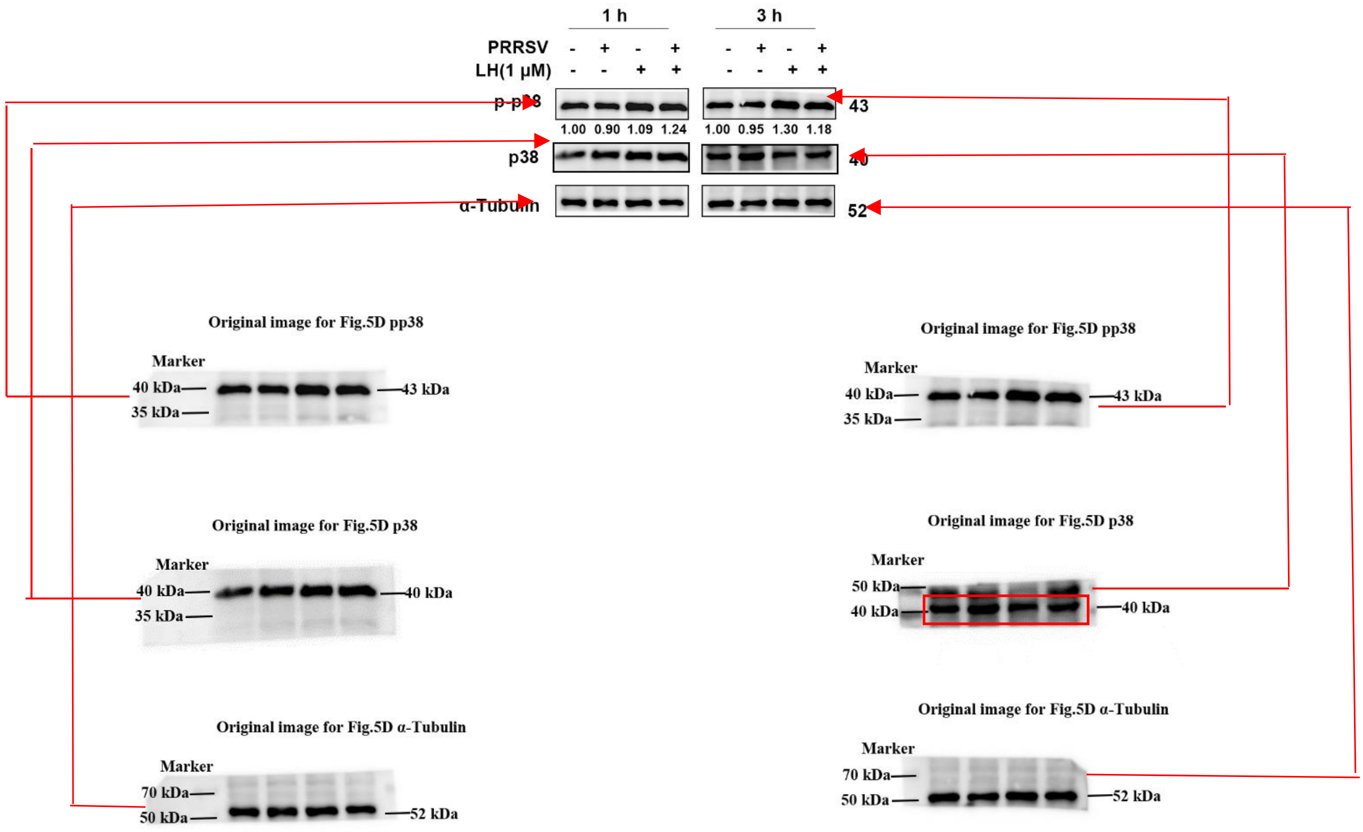

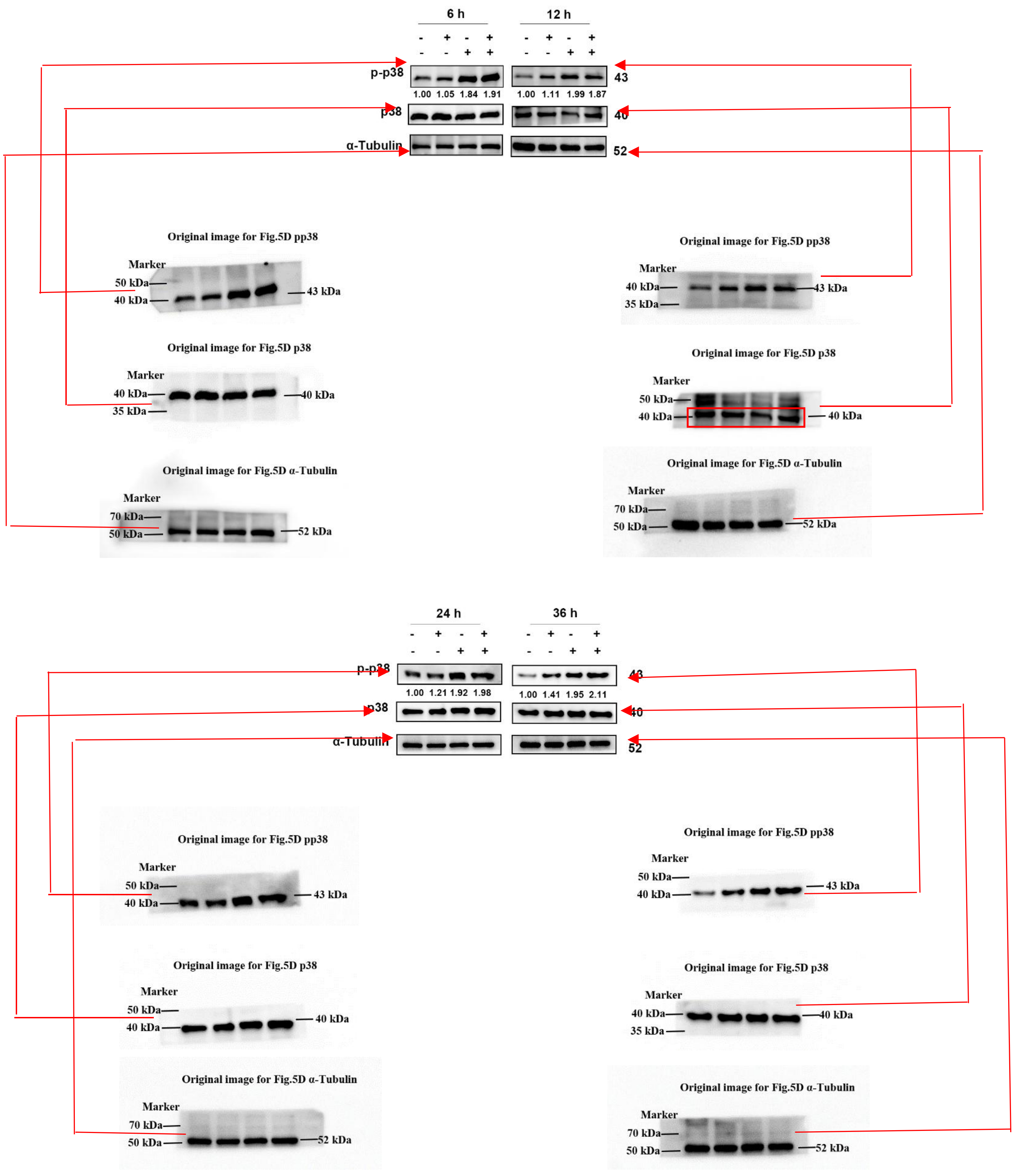
6 h
12 h
p-p38
43
1.00 1.05 1.84 1.91
1.00 1.11 1.99 1.87
p38
40
α-Tubulin
52
Original image for Fig.5D pp38
Marker
50 kDa
40 kDa
43 kDa
35 kDa
Original image for Fig.5D p38
40 kDa
Original image for Fig.5D α-Tubulin
70 kDa
50 kDa
52 kDa
24 h
36 h
1.00 1.21 1.92 1.98
1.00 1.41 1.95 2.11

Figure S7 The protein expression levels were detected by Western blot. (corresponding to Figure 6A)

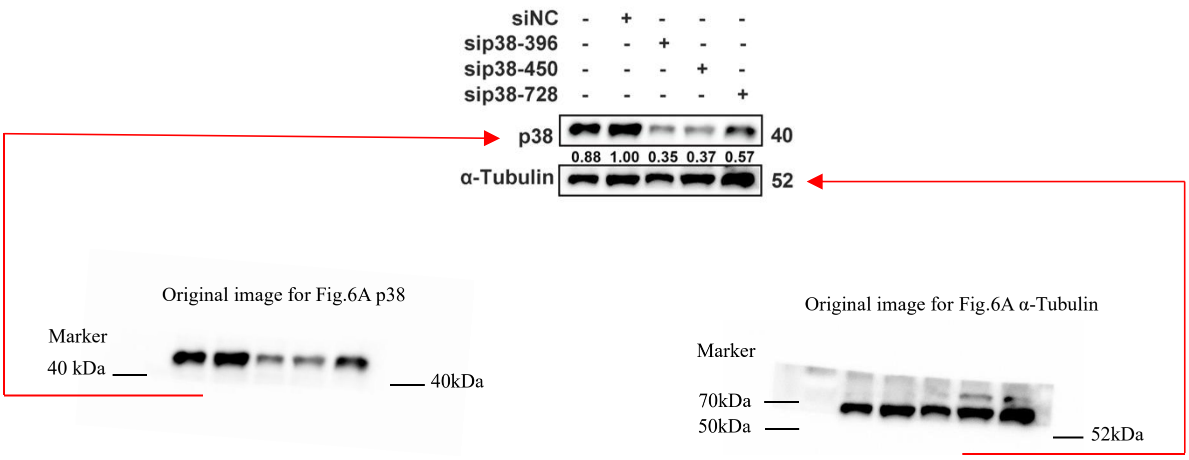

Figure S8 The protein expression levels were detected by Western blot. (corresponding to Figure 6B)

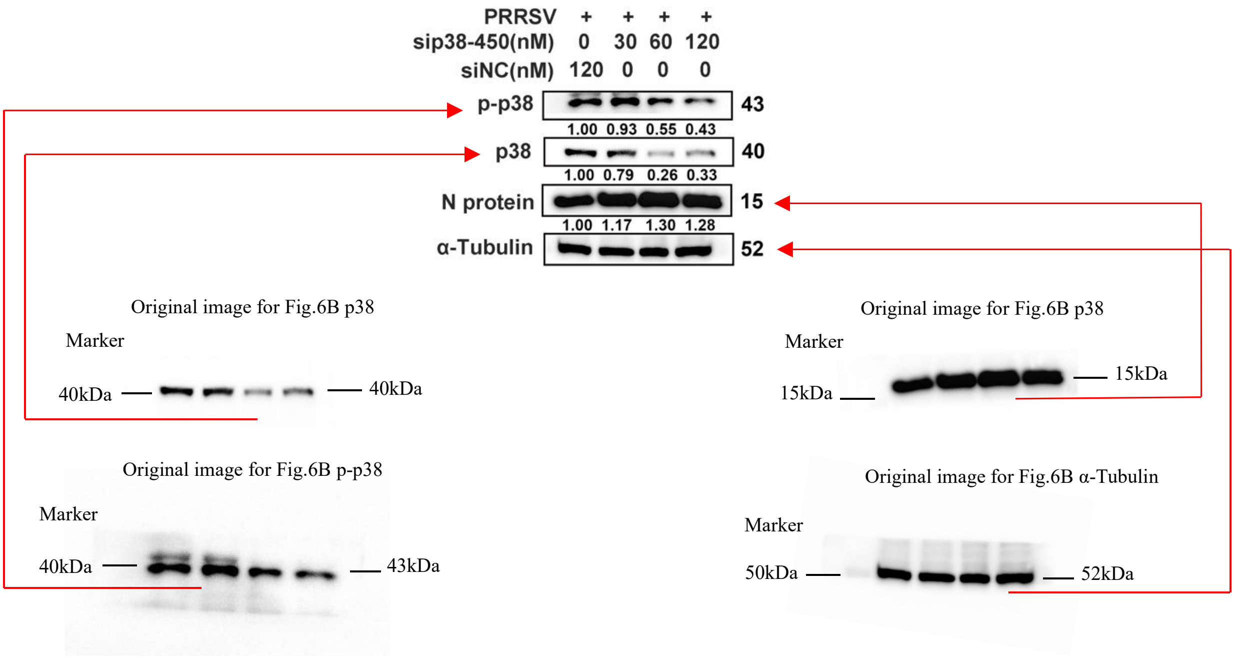

Figure S9 The protein expression levels were detected by Western blot. (corresponding to Figure 6C)

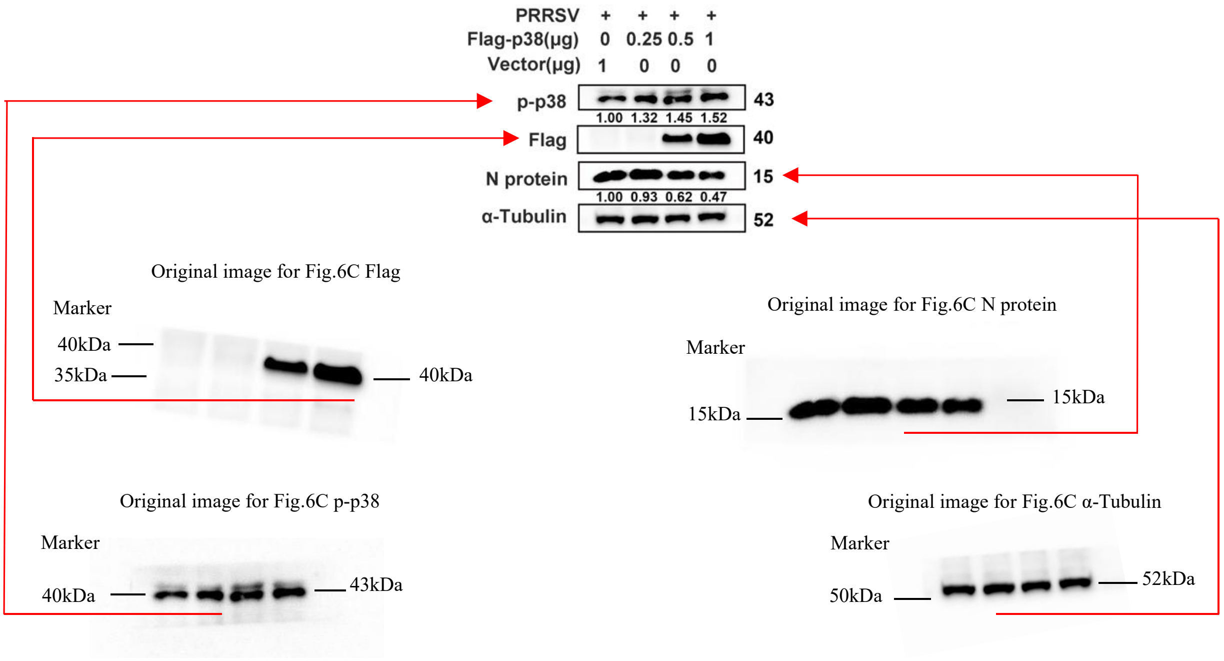

Figure S10 The protein expression levels were detected by Western blot. (corresponding to Figure 6D)

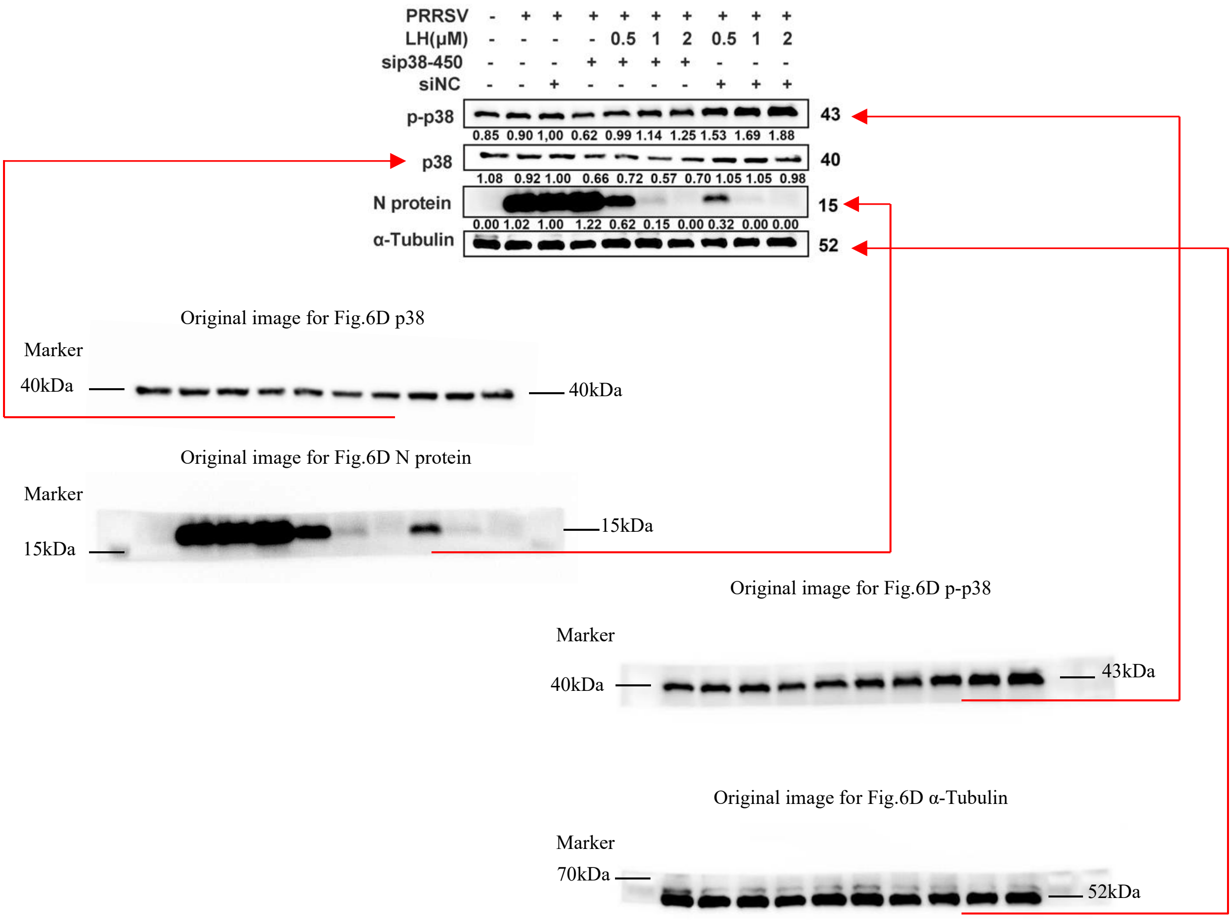

Figure S11 The protein expression levels were detected by Western blot. (corresponding to Figure 6E)

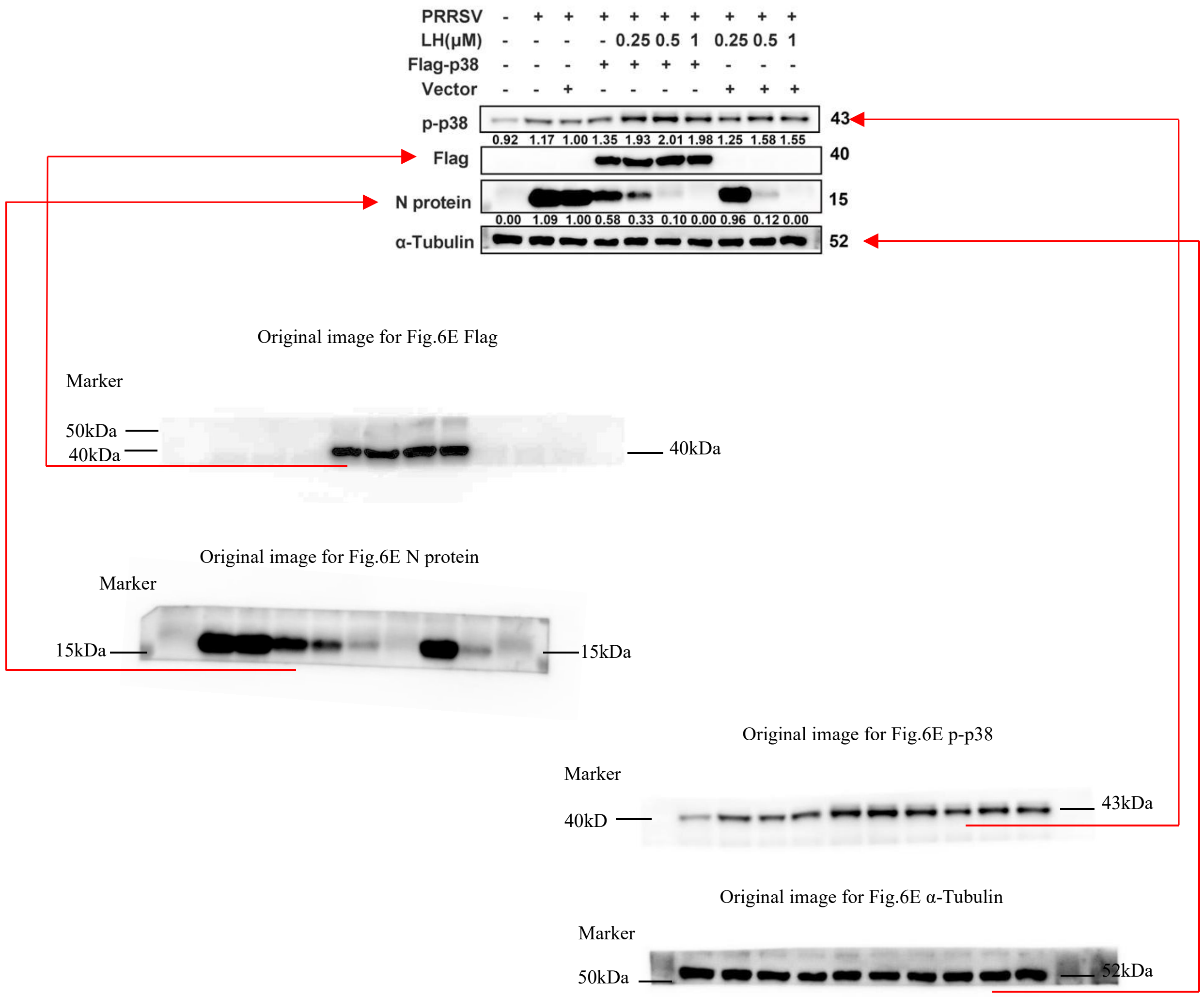

Figure S12 The protein expression levels were detected by Western blot. (corresponding to Figure 7A)

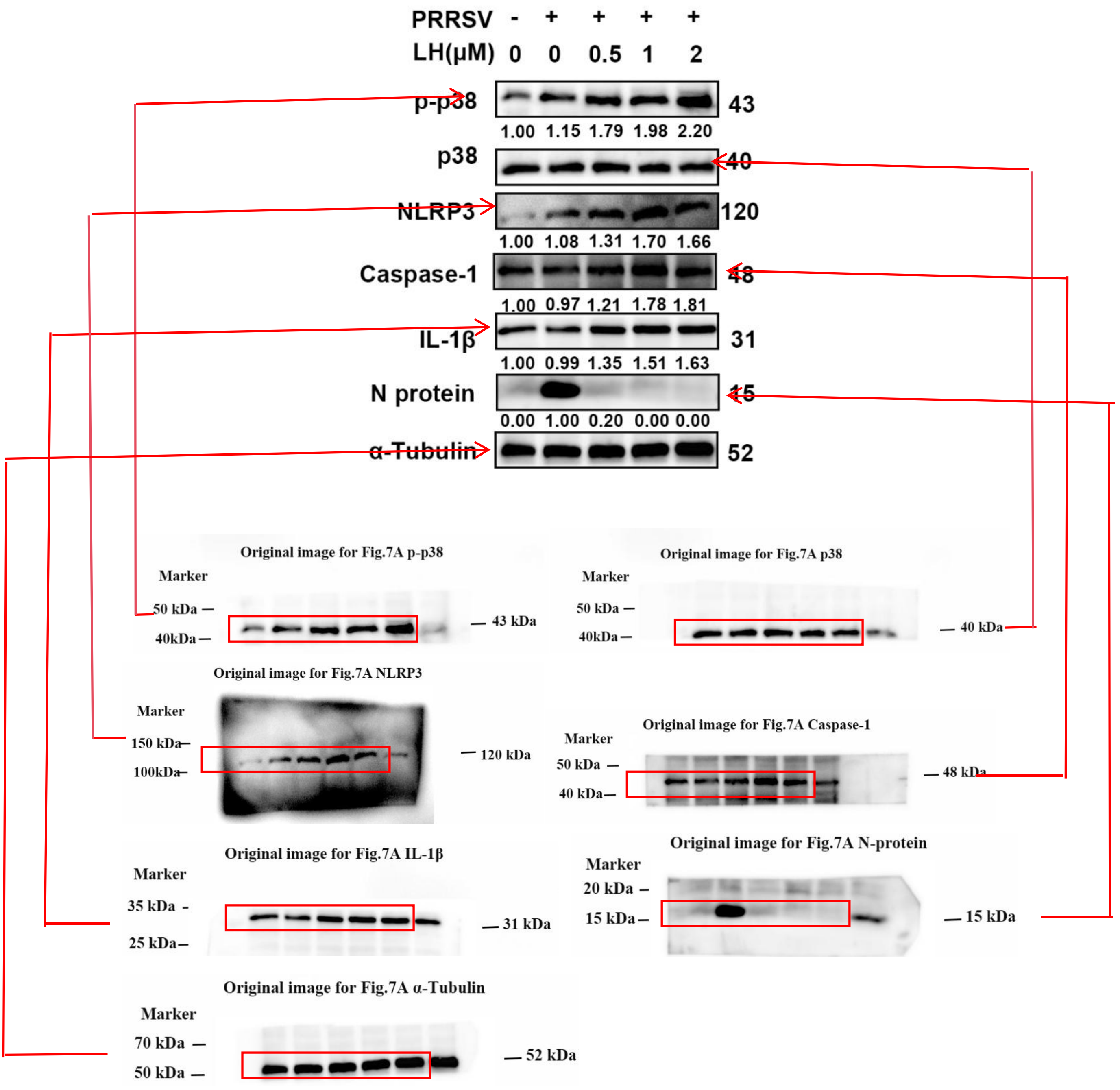

Figure S13 The protein expression levels were detected by Western blot. (corresponding to Figure 7B)

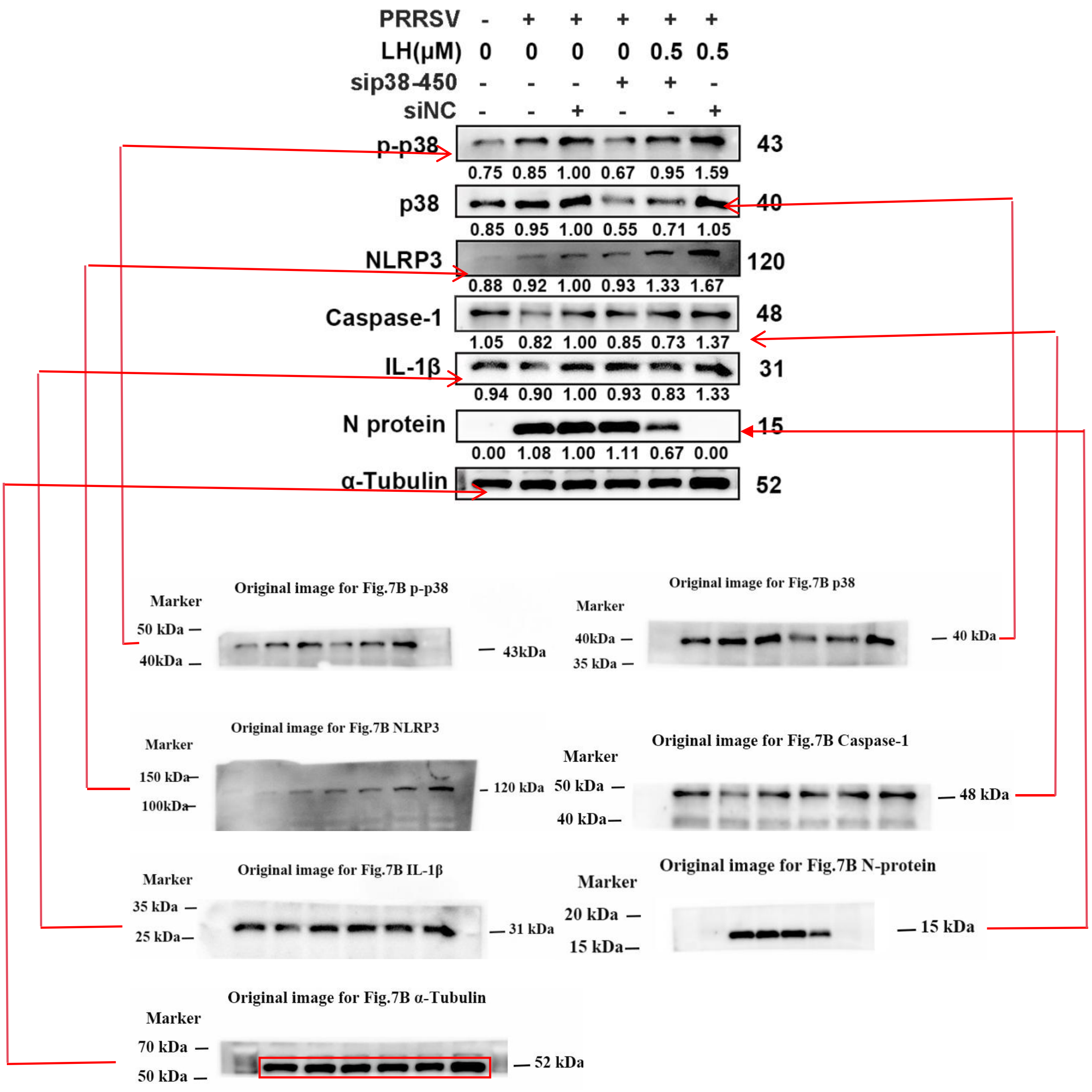

Figure S14 The protein expression levels were detected by Western blot. (corresponding to Figure 7C)

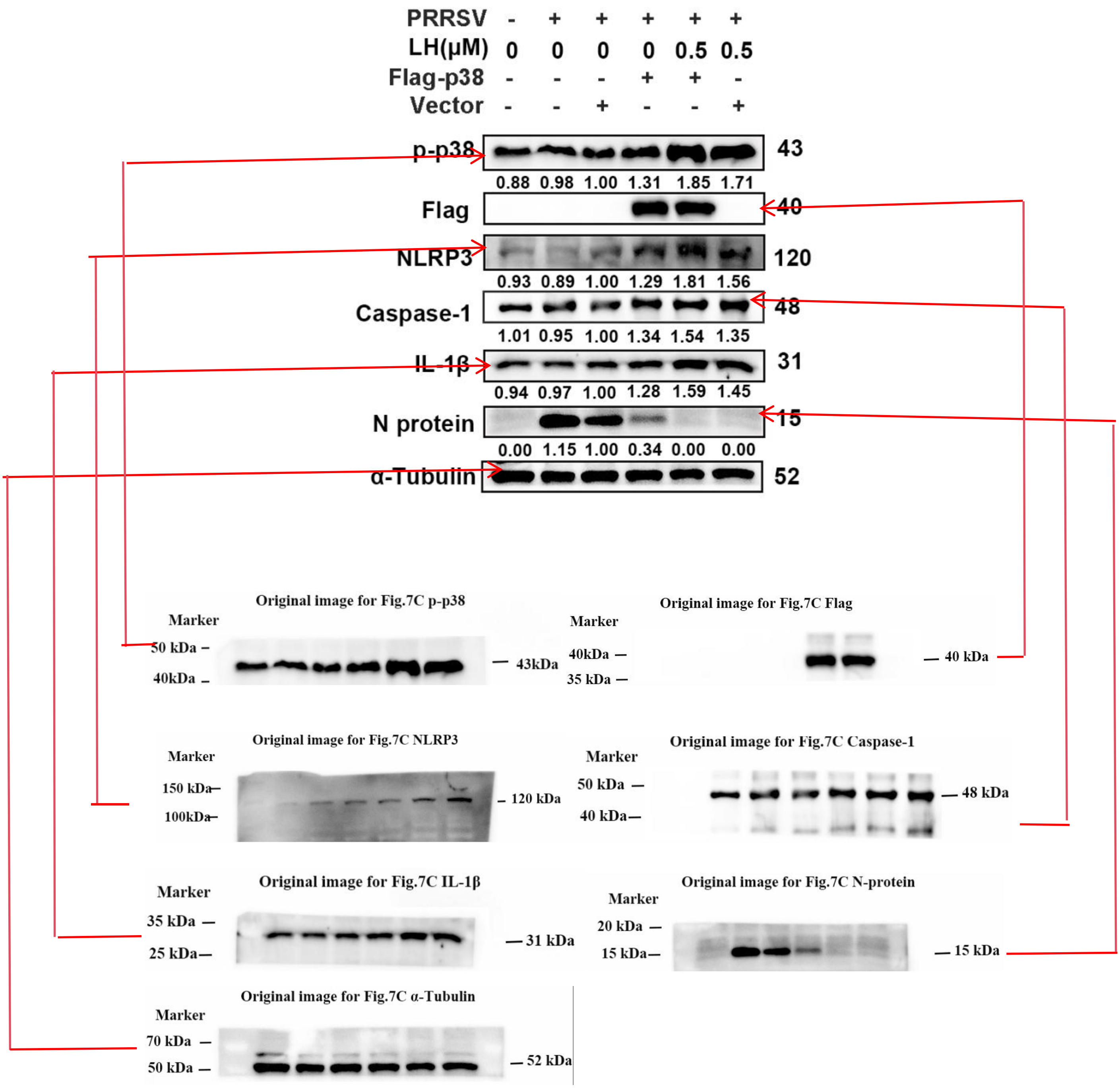

Figure S15 The protein expression levels were detected by Western blot. (corresponding to Figure7E)

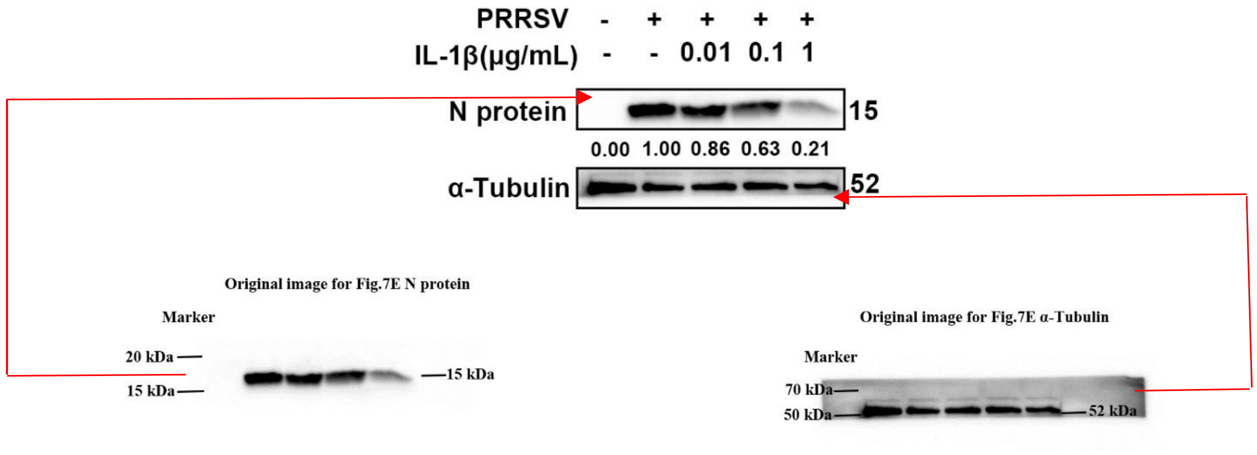

Figure S16 The protein expression levels were detected by Western blot. (corresponding to Figure 8C)

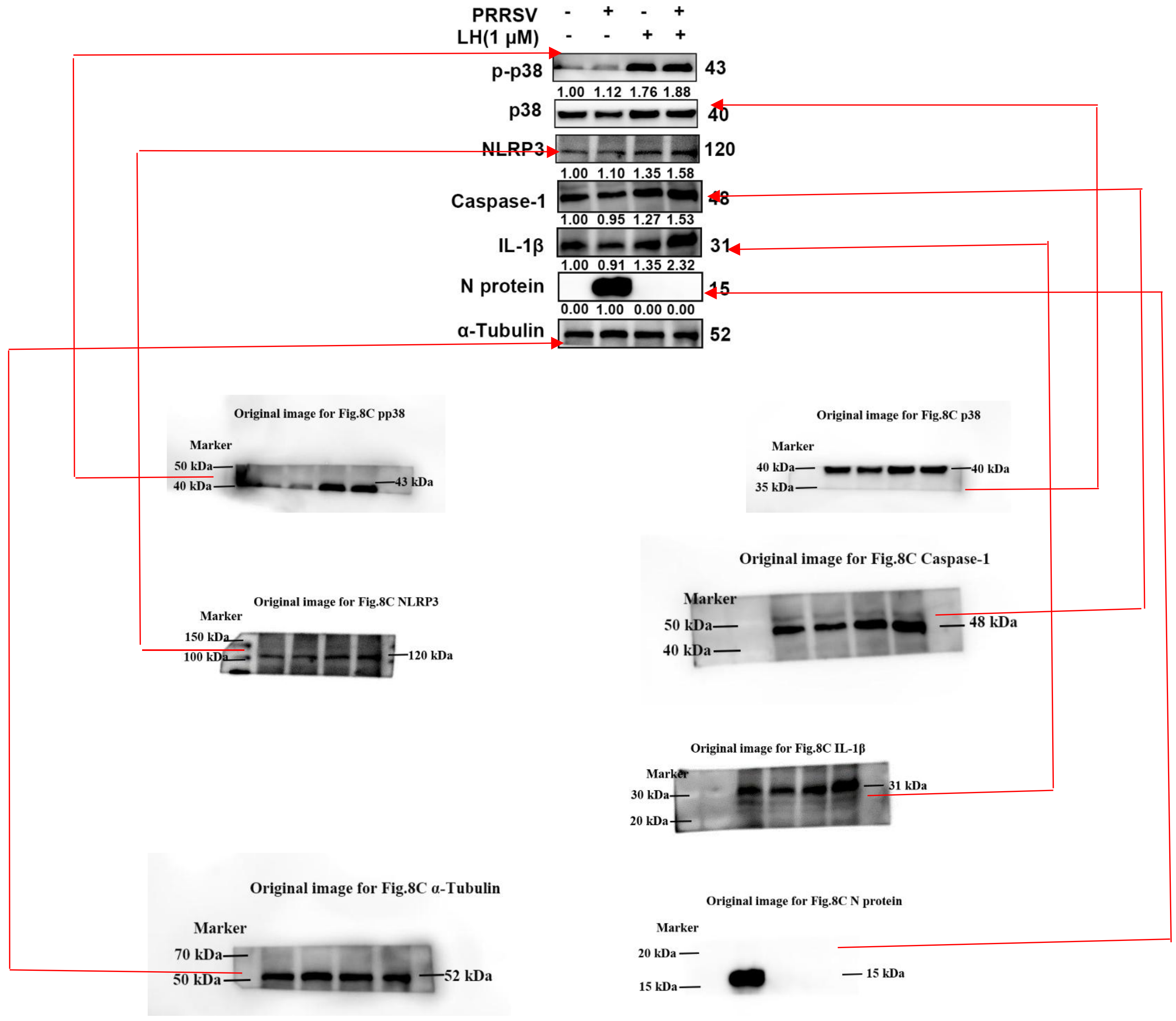

Figure S17 The protein expression levels were detected by Western blot. (corresponding to Figure8E)

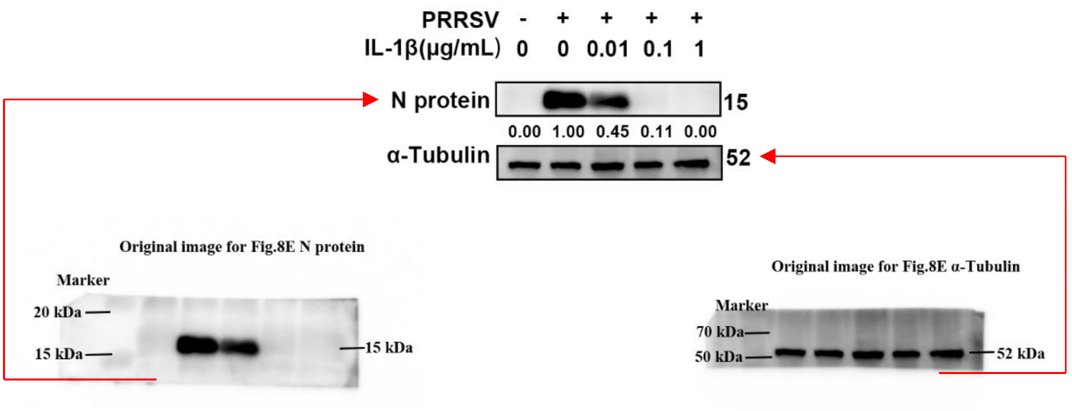

Figure S18 The protein expression levels were detected by Western blot. (corresponding to Figure S2)

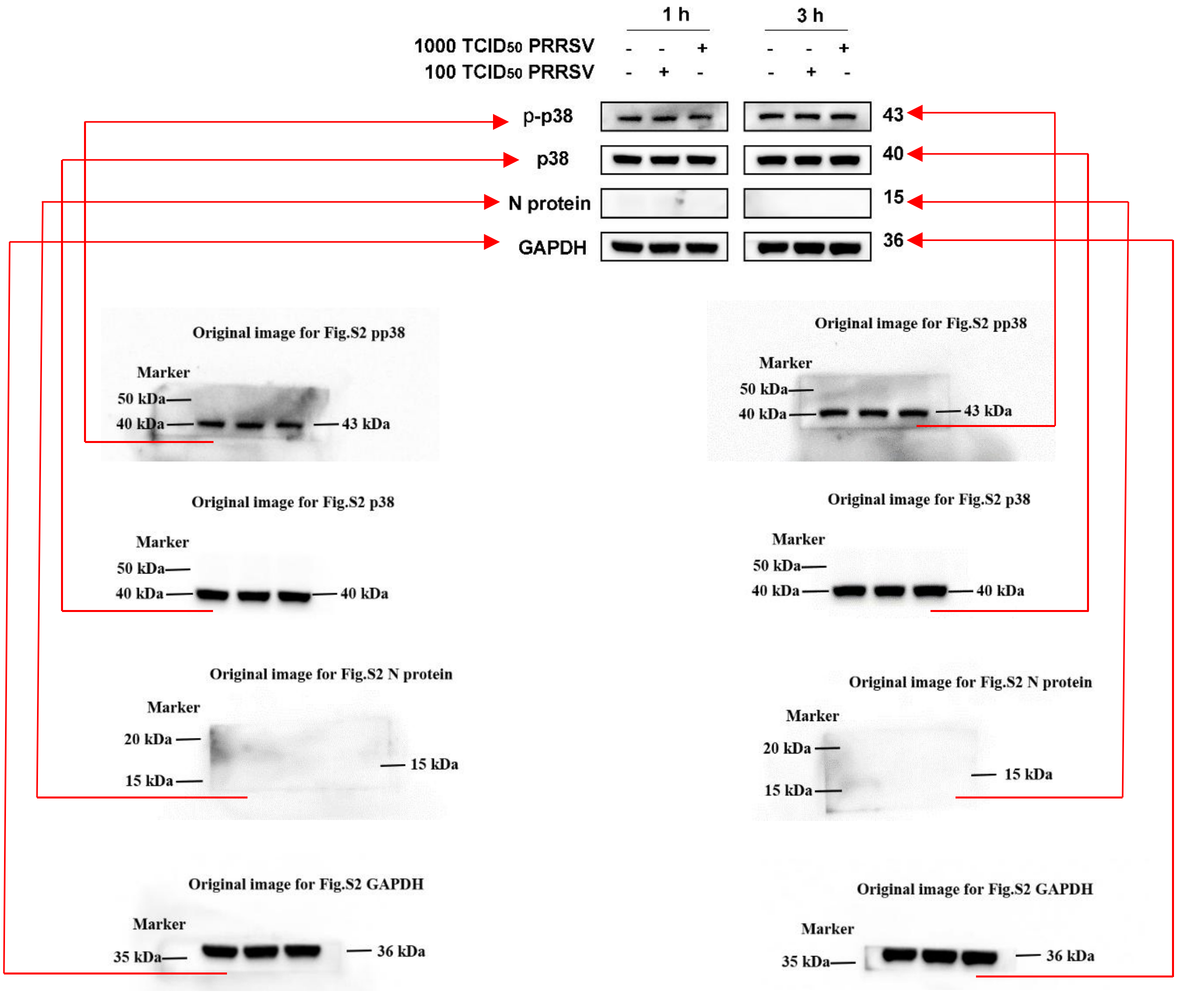

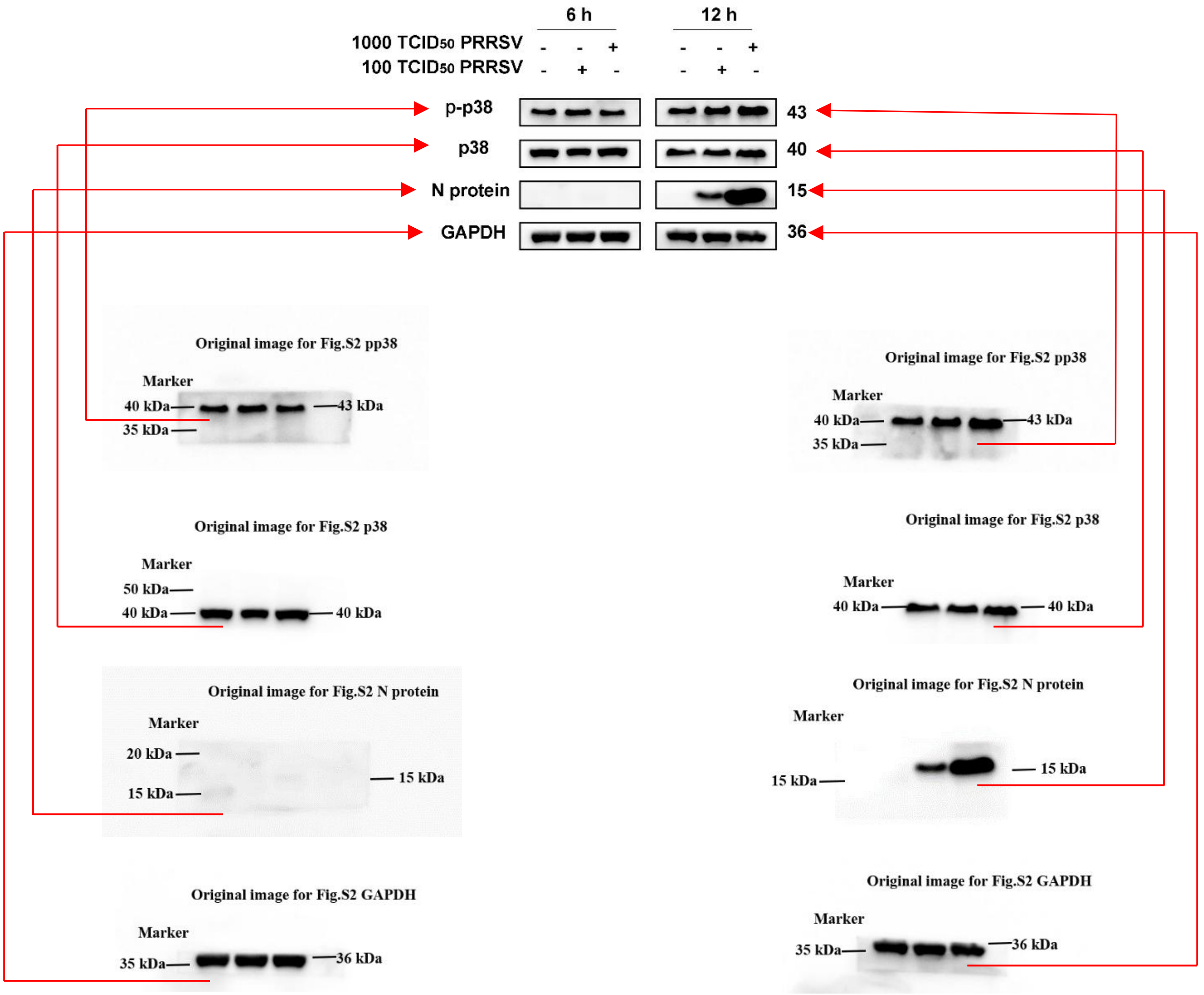

6 h
12 h
1000 TCID50 PRRSV
100 TCID50 PRRSV
p-p38
p38
N protein
GAPDH
43
40
15
36
Original image for Fig.S2 pp38
Marker
40 kDa
35 kDa
43 kDa
Original image for Fig.S2 p38
50 kDa
40 kDa
Original image for Fig.S2 N protein
20 kDa
15 kDa
Original image for Fig.S2 GAPDH
35 kDa
36 kDa

Figure S19 The protein expression levels were detected by Western blot. (corresponding to Figure S3)

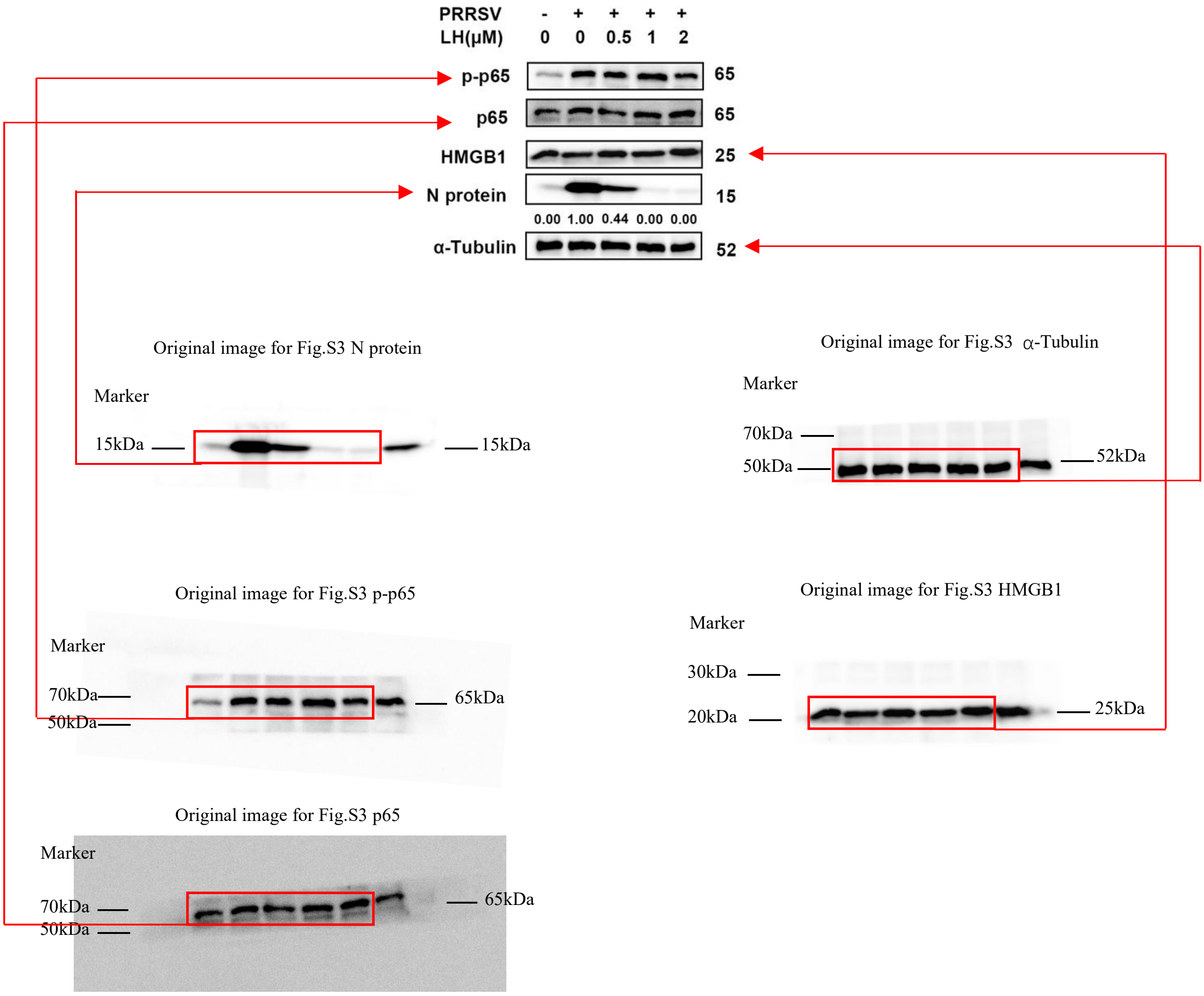

Shanghai Epizyme Biomedical Technology Co., Ltd, Multicolor Prestained Protein Ladder, CAT: WJ103, LOT: 027A10000
