# Supplementary material for: Early boosting of p38 MAPK signaling pathway by lycorine hydrochloride potently inhibits PRRSV proliferation in primary and established cells
Source: Front Microbiol. 2025 Aug 21;16:1664973. doi: 10.3389/fmicb.2025.1664973 (PMC12408688; doi:10.3389/fmicb.2025.1664973)
Supplement: Supplementary file 2 [file Data_Sheet_2.pdf]

Supplementary Material

Supplemental results

Figure S1. Inhibitory activity of Ribavirin against PRRSV replication in Marc-145 cells. (**A**) Antiviral activities of Rib against PRRSV GD-HD were examined by IFA. (**B**) The percentage of reduced PRRSV-infected cell number in IFA images was used to determine the concentration required to protect 50% cells from PRRSV infection (EC_{50}).

Figure S2. LH treatment does not directly inactivate PRRSV in Marc-145 cells. PRRSV GD-HD (1600 $TCID_{50}$) was mixed with 2 μM of LH in essential medium (1 mL total volume) for 2 h at 37℃. Then the mixture was 16-fold diluted, as a shown in (**A**). The mixture was resuspended to infect Marc-145 cells. After 48 h, the samples were analyzed by IFA (**B**).

Figure S3. PRRSV infections both at 100 and 1000 $TCID_{50}$ induce a late rather than an early activation of p38 MAPK. Marc-145 cells grown in 12-well plates were infected or mock-infected with PRRSV GD-HD (100 $TCID_{50}$ or 1000 $TCID_{50}$). The cells were collected at 1, 3, 6, 12 hpi, and total protein was extracted from cell lysates. The expression levels of p38 and p-p38 were analyzed by Western blotting.

Figure S4. LH treatment does not affect p-p65 expression in PRRSV-infected Marc-145 cells. Marc-145 cells were grown in 12-well plates were infected with PRRSV (100 $TCID_{50}$) for 2 h at 37℃, followed by culture in fresh medium containing various concentrations of LH. Total protein was extracted from cell lysates at 24 h and analyzed by Western Blotting.

Figure S5. Cytotoxicity of exogenous IL-1β in Marc-145 cells and PAMs. Cytotoxicity of exogenous IL-1β in Marc-145 cells (**A**) and PAMs (**B**) was evaluated using the MTT assay.
